# 3D-assembled microneedle ion sensor-based wearable system for the transdermal monitoring of physiological ion fluctuations

**DOI:** 10.1038/s41378-023-00497-0

**Published:** 2023-03-09

**Authors:** Xinshuo Huang, Shantao Zheng, Baoming Liang, Mengyi He, Feifei Wu, Jingbo Yang, Hui-jiuan Chen, Xi Xie

**Affiliations:** 1 State Key Laboratory of Optoelectronic Materials and Technologies, School of Electronics and Information Technology; Guangdong Province Key Laboratory of Display Material and Technology, Sun Yat-Sen University, Guangzhou, China; 2 Pazhou Lab, 510330 Guangzhou, China; 3 School of Biomedical Engineering, Sun Yat-Sen University, Guangzhou, China

**Keywords:** Electrical and electronic engineering, Biosensors, Biosensors

## Abstract

Monitoring human health is of considerable significance in biomedicine. In particular, the ion concentrations in blood are important reference indicators related to many diseases. Microneedle array-based sensors have enabled promising breakthroughs in continuous health monitoring due to their minimally invasive nature. In this study, we developed a microneedle sensing-array integrated system to continuously detect subcutaneous ions to monitor human health status in real time based on a fabrication strategy for assembling planar microneedle sheets to form 3D microneedle arrays. The limitations of preparing 3D microneedle structures with multiple electrode channels were addressed by assembling planar microneedle sheets fabricated via laser micromachining; the challenges of modifying closely spaced microneedle tips into different functionalized types of electrodes were avoided. The microneedle sensing system was sufficiently sensitive for detecting real-time changes in Ca^2+^, K^+^, and Na^+^ concentrations, and it exhibited good detection performance. The in vivo results showed that the ion-sensing microneedle array successfully monitored the fluctuations in Ca^2+^, K^+^, and Na^+^ in the interstitial fluids of rats in real time. By using an integrated circuit design, we constructed the proposed microneedle sensor into a wearable integrated monitoring system. The integrated system could potentially provide information feedback for diseases related to physiological ion changes.

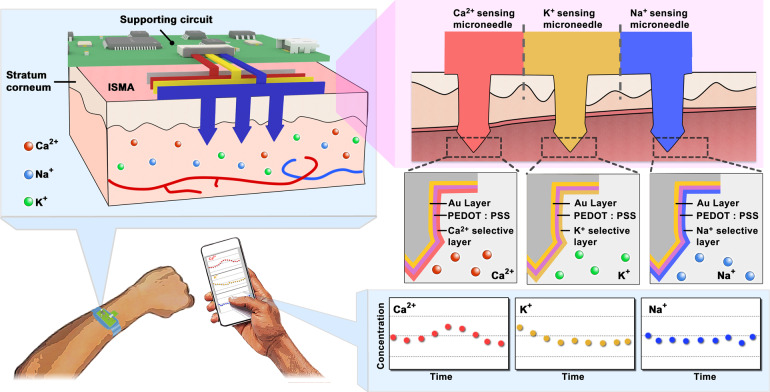

## Introduction

Human health monitoring and the early disease detection are important goals of modern medicine^1–3^. Monitoring long-term changes in human health makes early warning and intervention for many diseases possible; this monitoring provides an important reference for individual disease treatment^4–6^. Wearable health monitoring systems based on intelligent sensors can be attached to a human body, detect health features in situ in real time, and provide timely warning and feedback. In recent decades, scientists have made encouraging progress in basic research and clinical medicine with the emergence of new technologies, such as artificial intelligence and the Internet of Things^7–10^. Wearable health sensors can be comfortably attached to human bodies to record a series of health information, such as pulse rate, blood pressure, and glucose level^11,12^. Then, these data can be transmitted to intelligent devices to extract and analyze disease profiles^13,14^.

In recent years, various types of wearable sensors, such as wrist band-based sensors, sweat sensors, and contact lens-based sensors, have been widely reported for the noninvasive detection of human health information^4,15,16^. Gao et al. combined an integrated printed circuit board with flexible plastic substrate sensor technology; the researchers proposed a wearable flexible integrated sensing array for the simultaneous selective screening of biomarkers in sweat^17^. Duan et al. utilized conductive microstructured air gaps and integrated them with 2D semiconductor transistors to produce pressure sensors with ultrahigh sensitivity, providing an optional space for manufacturing a more extensible and high-sensitivity wearable medical treatment^18^. By combining strategic material integration and advanced micro/nanoprocessing, Xu et al. reported a new class of conformal and stretchable ultrasonic devices that may provide noninvasive, accurate, and continuous vital-sign monitoring through human skin^19^. Although these noninvasive sensors possess excellent biosafety, they cannot directly measure biochemical analytes within the body because they can only access substances on the skin surface^20,21^.

Recent measurement targets for these wearable sensors are mainly limited to physical signals, such as pulse rate, electrocardiogram (ECG), and physiological pressure, or biochemical molecules in secretions, such as sweat and tears^22–25^. Biochemical analytes in the body, such as ions (Ca^2+^, K^+^, Na^+^, pH) and metabolites (glucose, lactate, uric acid), are important indicators of the body’s health status; the measurements of these biochemical analytes from sweat or tears may significantly deviate from the actual concentrations in blood and interstitial fluids^21,26,27^. Although skin is the protective layer of the body, the stratum corneum hinders the detection of biochemical analytes in vivo by external devices. The ion concentration in the blood is an important reference index for many diseases. For example, the concentrations of sodium and calcium ions are closely related to hypernatremia and hypokalemia.

Interstitial fluid is a body fluid in the interstitial spaces of tissues; it is part of the internal cell environment, and the composition is basically the same as that of plasma without large molecules of protein. Low-molecular-weight analytes in interstitial fluid, including metabolites (glucose and lactate) and electrolytes (Na^+^, K^+^), are close to the plasma concentration^16,28–31^. The recent method for measuring ion concentration includes blood sampling through metal needles, which leads to risks of pain and infection due to the trauma caused by needles^24,32–34^. In addition, blood sampling can only be performed by trained technicians in hospitals, which hinders the long-term, repeated and continuous monitoring of ion fluctuations in the body^35,36^. Due to the rapid development of micro/nanofabrication technologies, microneedles of 500–800 μm lengths that can penetrate the stratum corneum of the skin and access the interstitial fluid without reaching blood vessels or nerves within the dermis have attracted much attention^37,38^. The microneedles not only avoid pain but also reduce local inflammation or fibrosis caused by tissue trauma. For example, soluble microneedle patches for drug delivery have been successfully applied to treating high blood pressure, tumor diseases, and cardiovascular diseases in recent years^39–41^. Moreover, microneedle-array-based sensors have achieved promising breakthroughs in continuous blood-glucose monitoring^42–44^. However, there are few wearable devices used for ion monitoring based on microneedle arrays, especially for monitoring different types of ions (e.g., Na^+^, K^+^, Ca^2+^) simultaneously; this phenomenon occurs because the 3D structure of the microneedle array is more demanding for manufacturing different types of sensors in microneedle arrays than for processing planar electrodes and for changing the functionalization on closely spaced microneedle tips^27,45–47^.

**Fig. 1:**
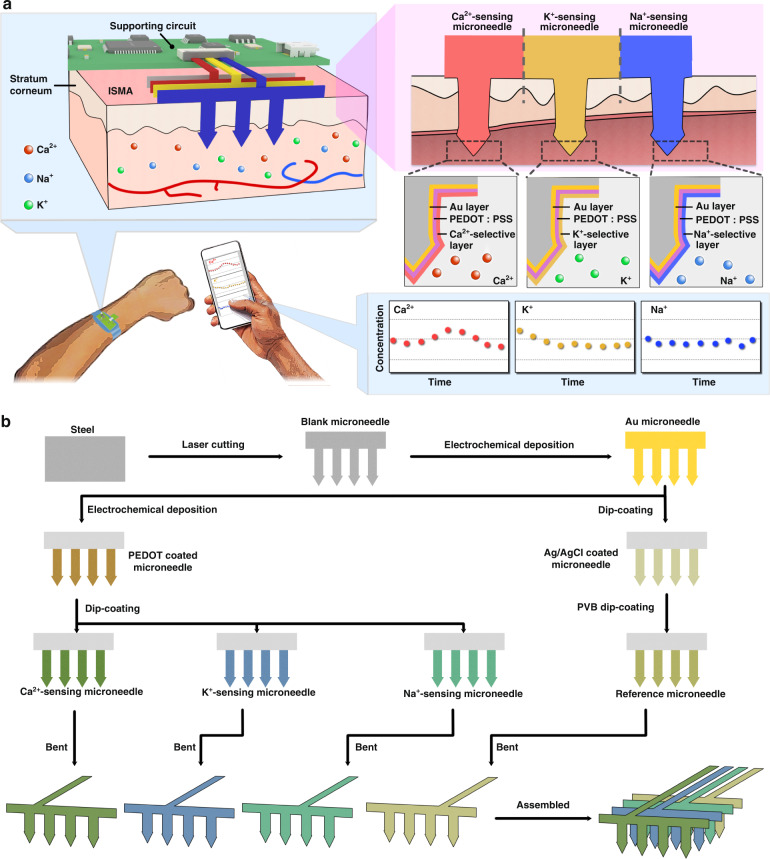
Schematics and fabrication process of the microneedle sensor for continuous ion monitoring. **a** Schematic of the microneedle-array sensor for ion detection. The microneedle-array sensor, which can be worn on the hand, performs minimally invasive detection through the skin to measure the concentrations of different ions in the interstitial fluid. The sensor transmits the signal through a printed circuit board to a microprocessor for signal processing and display. **b** Schematic of the fabrication method of the microneedle-array sensor. The planar sheet is processed into a planar microneedle structure via laser micromachining, and a conductive Au layer and PEDOT layer are electrochemically deposited on the microneedle tips; afterward, the ion-selective film is modified on it. These form the microneedle working electrodes, which achieve the selective detection of ions. Moreover, the Au-coated microneedles are further modified with Ag/AgCl ink and a PVB protective layer to form a microneedle reference electrode. The three microneedle patches (working electrodes for Ca^2+^, K^+^, and Na^+^ sensing) are integrated with the microneedle reference electrode to obtain the ion-sensing microneedle array (ISMA). The ISMA can be further integrated with a printed circuit board to form an integrated system, which supports the sensing functionalities of the microneedle device

In this study, we have developed a microneedle sensing-array integrated system for the continuous detection of subcutaneous ions in real time based on a fabrication strategy for assembling planar microneedle sheets to form 3D microneedle arrays, as shown in Fig. 1a. The limitations of manufacturing 3D microneedle structures with multiple electrode channels and the challenges of modifying closely spaced microneedle tips into different functional types of electrodes are overcome by assembling planar microneedle chips manufactured by laser micromachining. The system consists of three interconnected modules: (1) an ion-sensing microneedle array, (2) a printed circuit board as a supporting part for recording and control, and (3) a mobile application for real-time monitoring. As the key component, the ion-sensing microneedle array can penetrate the stratum corneum painlessly to detect real-time changes in Ca^2+^, K^+^, and Na^+^ concentrations in subcutaneous tissue fluid in real time. The system may serve as an important tool for the real-time monitoring of human health and may potentially provide information feedback for diseases related to physiological ion changes.

## Results and discussion

### Fabrication of ISMA

The ion-sensing microneedle array (ISMA) was constructed with three parallel electrodes to detect Ca^2+^, K^+^, and Na^+^ to prepare a microneedle sensor that can simultaneously detect multiple ions. The three working electrodes of the sensor shared the same reference electrode, and all four electrodes needed to be insulated from each other. Ion-sensing microneedle electrodes were based on a potentiometric sensing mechanism that employed a two-electrode system to measure the signal of the potential change associated with ion concentrations. By coating the microneedle electrode surface with an ion-selective membrane, the target ion was allowed to pass through the ion-selective membrane, reaching the electrode surface underneath the membrane; nontarget ions could not reach the electrode due to the barrier of the membrane^48^. Ion collection generated a potential difference between the microneedle working electrode and the reference electrode in solution according to the target ion concentration.

The assembly of microneedle arrays from flat microneedle sheets posed a challenge because of the following problems: (1) microneedles based on stainless steel have high rigidity, but the presence of impurities hinders the electrochemical properties of the prepared electrode and (2) due to the need for heating during the welding process, microneedles can only be used to detect a single biomarker; this phenomenon affects the modified sensing film layer, leading to wide applicability in drug delivery and less applicability for sensing and detection^49,50^. To address these issues, we extended this technique and innovated the integration method, enabling multiparameter sensing that was more sophisticated than the previous single-parameter method.

Each electrode was based on a planar microneedle sheet structure, which was subsequently assembled between the polydimethylsiloxane (PDMS) layers that were utilized as insulating layers. The fabrication process of the microneedle array and sensor device is shown in Fig. 1b. First, the structural pattern of the microneedle sheet was designed and prepared via laser micromachining on planar stainless steel sheets (Fig. S1, Supplemental Material). Each microneedle sheet contained five microneedle tips, each of which had thicknesses of 200 µm, lengths of 1200 µm, base diameters of 440 µm, and sharp structures at the tops of their microneedle. The microneedles had substrate widths of 3 mm and a stainless steel strips (length: 15 mm) as the conductive lead. We used electrochemical deposition with a −0.8 V voltage applied in an Au sulfite solution to prepare an Au layer on the stainless steel microneedle tip to improve the corrosion resistance, biocompatibility, and stability characteristics of the electrodes. For the working electrode used for ion detection, we further applied 1.1 V in the poly(3,4-ethylenedioxythiophene) polystyrene sulfonate (PEDOT:PSS) solution and prepared PEDOT on the surface of the microneedle tip. This action further improved the electron transfer performance and the stability of the microneedle electrode in solution. With improved mechanical robustness, the PEDOT layer enhanced the adhesion of the polymeric ion-selective membrane on the electrode to effectively prevent membrane peeling from the Au electrode surface.

For the microneedle working electrodes, the microneedle electrode tips were modified with Ca^2+^, K^+^, and Na^+^-selective membranes via dip coating. For the reference electrode, the surface of the microneedle electrode was modified with a Au layer via electrodeposition, followed by the application of −0.85 V in a silver sulfite solution to further electroplate the Ag layer. After treatment with HCl solution, an Ag/AgCl-coated electrode was formed; the electrode was further modified on the surface with polyvinyl butyral (PVB) via dip coating to slow the oxidation of Ag and improve the stability of the ion-sensing reference electrode. The reference electrode prepared by this method could maintain a stable reference potential that was almost immune to changes in the chloride concentration in solution (Fig. S2, Supplemental Material); this phenomenon improved the stabilities of the detection signals of the microneedle electrochemical system in subcutaneous interstitial fluids.

**Fig. 2:**
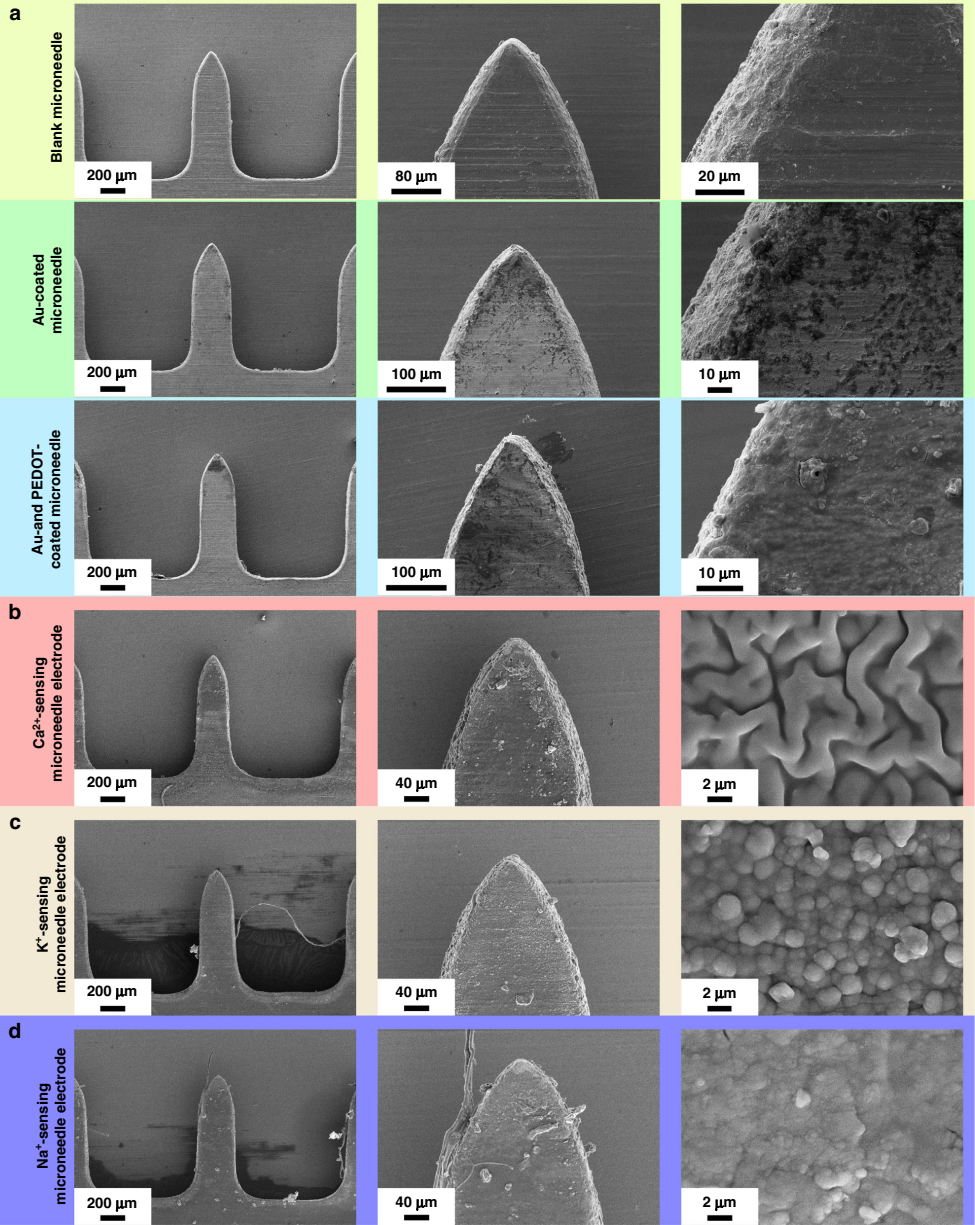
Morphological characterization of the blank microneedle patch, microneedle patch modified with the Au layer, microneedle patch modified with the PEDOT layer, and microneedle patch modified with ion-selective layers. **a** From left to right: blank steel microneedles, blank microneedles modified with Au layer, microneedles modified with the PEDOT ion conversion layer, and SEM images at different magnifications. **b**–**d** Optical and SEM characterizations of microneedle ion electrodes: **b** Ca^2+^-sensing microneedle electrode, **c** K^+^-sensing microneedle electrode, and **d** Na^+^-sensing microneedle electrode

Finally, the conductive leads on the substrates of the electrodes were bent and arranged between 3 mm-thick PDMS layers, bonded, and assembled as a 5 mm-thick 3D microneedle device with a tip spacing of 3 mm. The three working electrodes corresponded to detect Ca^2+^, K^+^, and Na^+^. This method required planar microneedles to be assembled on a support with a specific structure, and it could increase the complexity and cost of the manufacturing process; however, the method had the potential to be developed as a simple technique for manufacturing microneedle electrode arrays that reduce the complexities of functional 3D devices. The surface morphologies of the microneedle electrode sheets during the preparation process were characterized using microscopy and scanning electron microscopy (SEM). As shown in Fig. 2, the stainless steel microneedle sheet and Au microneedle sheet after Au plating possessed a smooth and uniform surface morphology; the microneedle electrode sheet after PEDOT modification (Fig. 2a) and after being coated with an ion-selective membrane displayed a corresponding polymer layer morphology on the surface (Fig. 2b–d).

### In vitro test of ISMA

**Fig. 3:**
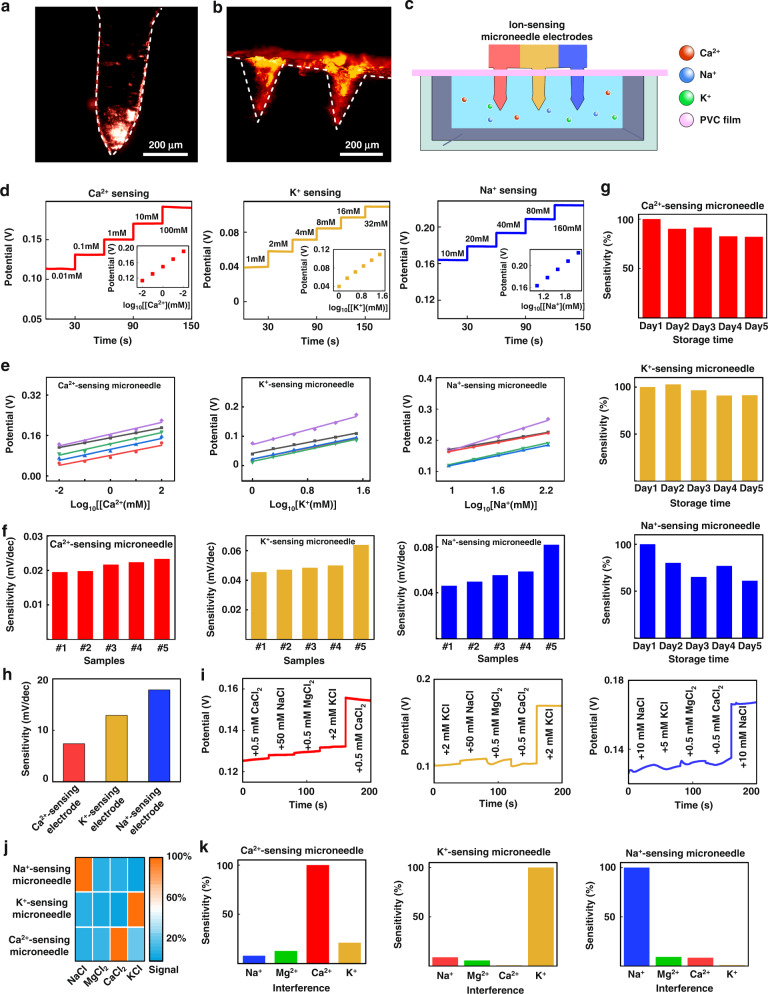
Response performance and detection selectivity characteristics of the ion-sensing microneedle electrodes to different ions. **a** Fluorescence images of the ion-sensing microneedle electrode stained with rhodamine B. **b** Fluorescence image showing rhodamine B deposition into porcine skin by ion-sensing microneedle electrode penetration. **c** Schematic showing the experimental setup of the ion-sensing microneedle electrode for continuous monitoring in vitro. **d** Diagram showing the linear responses of ion-sensing microneedle electrodes to different concentrations of Ca^2+^, K^+^, and Na^+^. **e**, **f** Parallel experiments on the linear responses of the ion-sensing microneedle electrodes to detect different concentrations of Ca^2+^, K^+^, and Na^+^. **g** Statistical analysis of the relative stabilities of the linear responses using the ion-sensing microneedle electrodes to detect different concentrations of Ca^2+^, K^+^, and Na^+^ over 5 days. **h** Ion-sensing microneedle electrode sensitivities to detect Ca^2+^, K^+^, and Na^+^. **i**–**k**: **i** Specific responses of the ion-sensing microneedle electrodes to Ca^2+^, K^+^, and Na^+^ with relative changes in the response voltage (absolute values). **j** Heatmap showing the specific responses of the ion-sensing microneedle electrodes to Ca^2+^, K^+^, and Na^+^ with relative changes in the response voltage (relative value). **k** Comparison of the interference resistance levels of the ion-sensing microneedle electrodes to Ca^2+^, K^+^, and Na^+^

The penetration ability of the microneedle was characterized in vitro using porcine skin instead of human skin. First, a blank microneedle tip was stained with red fluorescent rhodamine B; afterward, the patch was pressed onto the porcine skin by applying a positive force from top to bottom perpendicular to the fresh porcine skin surface. After standing for 3 min, the microneedle was removed, and the skin was observed under fluorescence microscopy (Fig. 3a). The surface of the treated skin retained a series of red fluorescence signals (Fig. 3b), indicating that the microneedles penetrated the skin successfully. The optical and fluorescence patterns of the top and cross-sectional views of the porcine skin transdermal area indicated distinct penetration holes. As shown in the figure, the maximum penetration depths of the microneedle holes were approximately 250 μm; these depths were shorter than the microneedle tip lengths (~680 μm). We speculated that this result occurred due to the good elasticity of porcine skin. The porcine skin produced contraction, which in turn caused the bottom of the tip hole to shrink or caused the tip to not penetrate completely into the bottom of the skin. However, the average penetration depths of the microneedle tips were 200–300 μm; these depths were deeper than the overall thicknesses of the entire stratum corneum (10–15 μm) and epidermis (50–100 μm) layers of the human body. Therefore, the microneedle tips could penetrate the dermis, which indicated good penetration characteristics.

The prepared ion-sensing microneedle electrodes were tested in vitro. As shown in Fig. 3c, a polyvinyl chloride (PVC) film was used to simulate the skin layer; the film was placed on top of the testing solution. The microneedle electrode penetrated the polymer film, and the electrode tips accessed the solution below it for sensing. Typically, the ion concentrations in the human body fell in the following ranges: 133–146 mmol/L for Na^+^, 3.2–5.7 mmol/L for K^+^, and 1.84–2.72 mmol/L for Ca^2+^.^31^ The in vitro tests used wider concentration ranges that included the above in vivo ion concentration ranges to prove the wider applicability of the as-prepared microneedle sensor. For example, Na^+^ was tested from 5 mmol/L to 160 mmol/L, K^+^ was tested from 1 mmol/L to 32 mmol/L, and Ca^2+^ was tested from 0.01 mmol/L to 100 mmol/L. To sense Ca^2+^ using a microneedle, gradient concentrations of Ca^2+^ ranging from 0.01 mmol/L to 100 mmol/L in aqueous solution were tested in vitro. The Ca^2+^-selective membrane selectively permitted the passage of Ca^2+^ through the membrane to reach the electrode surface inside the membrane; other ions could not reach the electrode due to the membrane barrier. A concentration difference was generated between the concentration of Ca^2+^ inside the membrane and that in the external environment, which produced a particular electric potential difference. The potential difference between the microneedle working electrode and reference electrode of the sensor was measured and correlated with the corresponding ion concentration. The results showed that as the Ca^2+^ concentration gradually increased from 0.01 mmol/L to 100 mmol/L, the potentiometric signal of the electrode increased gradually from 69 mV to 155 mV. A linear fit between the voltage signal and the Ca^2+^ concentration was performed, and the results (Fig. 3d) revealed that the potentiometric signal of the microneedle electrode possessed good linearity with Ca^2+^ concentration; there was an average detection sensitivity of 21.65 mV/degree (*R*^2^ = 0.9450). Similarly, the concentration of K^+^ gradually increased from 2 to 32 mmol/L, and the potential signal of the microneedle electrode increased gradually from 2 to 91 mV. Moreover, the voltage signal of the microneedle electrode had a good linear correlation to the concentration of K^+^ with an average sensitivity of 47.1 mV/degree (*R*^2^ = 0.9952). When sensing Na^+^, their concentration gradually increased from 5 to 160 mmol/L; the potential signal of the microneedle electrode gradually increased from 152 to 268 mV. Similarly, the voltage signal of the microneedle electrode exhibited good linearity with the concentration of Na^+^ with an average sensitivity of 76.24 mV/degree (*R*^2^ = 0.9814).

The reproducibility of different microneedle electrodes was evaluated to facilitate the mass production of ISMA and to verify their potential for large-scale applications. Five batches of the different ion-sensing microneedle electrodes (Ca^2+^, K^+^, and Na^+^) were tested in the test solution by preparing them in parallel. As shown in Fig. 3e, a comparison of the responses of several ion-sensing microneedle electrodes produced in the different batches revealed that with increasing substrate concentration, sensors of the same type presented similar trends and operated with similar sensitivities (approximately 20% error). Notably, the absolute potential differences between different production batches of the same type of sensor should not be ignored; the response variations for each electrode group are further illustrated in Fig. 3f, h. The relative standard deviations (SDs) for Ca^2+^, K^+^, and Na^+^ detection using microneedle electrodes were 7.4, 12.9, and 17.8%, respectively; these results demonstrated the stability and reproducibility characteristics of the ion-sensing microneedle electrodes.

To facilitate the usage of the electrodes in real-life situations, the stabilities of the ion-sensing microneedle electrodes were investigated over time, and the preservation conditions and effects were explored. Figure 3g shows the daily sensitivity changes in each microneedle sensor within 5 days; the sensitivity change for each subsequent day was calibrated by testing different electrodes and setting the sensitivity on the first day to 100% as the base value. The final relative sensitivities of these sensors after four days were 81.98, 91.25, and 61.00% for the Ca^2+^-, K^+^-, and Na^+^-sensing electrodes, respectively. The results suggested that the Ca^2+^-sensing and K^+^-sensing electrodes maintained excellent temporal stability; however, the Na^+^-sensing electrodes exhibited significant changes in sensitivity that were difficult to ignore from day two onward. The absolute sensitivities of these sensors are displayed in Fig. S3 (Supplemental Material). These results revealed that the sensitivities of the ion-sensing microneedle electrodes remained usable over four days of storage, thereby demonstrating the importance of precalibration to improve the stabilities of the sensors.

After integrating microneedle electrodes into an ion-sensing microneedle array, the coexistence of multiple markers for detecting individual ions could interfere with the accuracy of the detection. Therefore, the activities of the microneedle electrodes were evaluated using the corresponding markers. Figure 3i–k depicts the responses of these different electrodes (Ca^2+^, K^+^, and Na^+^) to the target and interfering substances, respectively, under continuous monitoring. As shown in Fig. 3i, the presence of 0.5 mmol/L Ca^2+^ produced a signal reaching 35 mV for the Ca^2+^ sensor, while the addition of interfering substances produced a weak signal (4 mV). Similarly, for the K^+^ sensor, the addition of 2 mmol/L K^+^ produced a signal reaching 65 mV; additions of interfering substances produced weak signals (<7 mV). For the Na^+^ sensor, the addition of 10 mmol/L Na^+^ produced a signal reaching 38 mV; additions of interfering substances produced weak signals (<6 mV).

To directly demonstrate the responses of the individual ion electrodes to interfering substances, the response currents of the electrodes to different interfering substances were normalized and quantified, as shown in Fig. 3j and Fig. S4 (Supplemental Material). From the above results, it was clear that for the microneedle array sensor, each interfering substance produced no more than 10% of the interference signal; this phenomenon indicated that the microneedle array sensor could simultaneously achieve the continuous monitoring of multiple ions in complex environments, such as tear, sweat, or interstitial fluids. To maintain the accuracy of continuous monitoring, the data acquisition time of the ion-sensing electrode was set to 60 s; the average current value obtained during the last 10 s was regarded as the final response value for the calculation. As shown in Fig. 3k, the three electrodes (Ca^2+^, K^+^, and Na^+^) that were integrated into the microneedle array provided excellent responsiveness for the simultaneous monitoring of complex analytes when connected to the circuit.

### In vivo test of ISMA

**Fig. 4:**
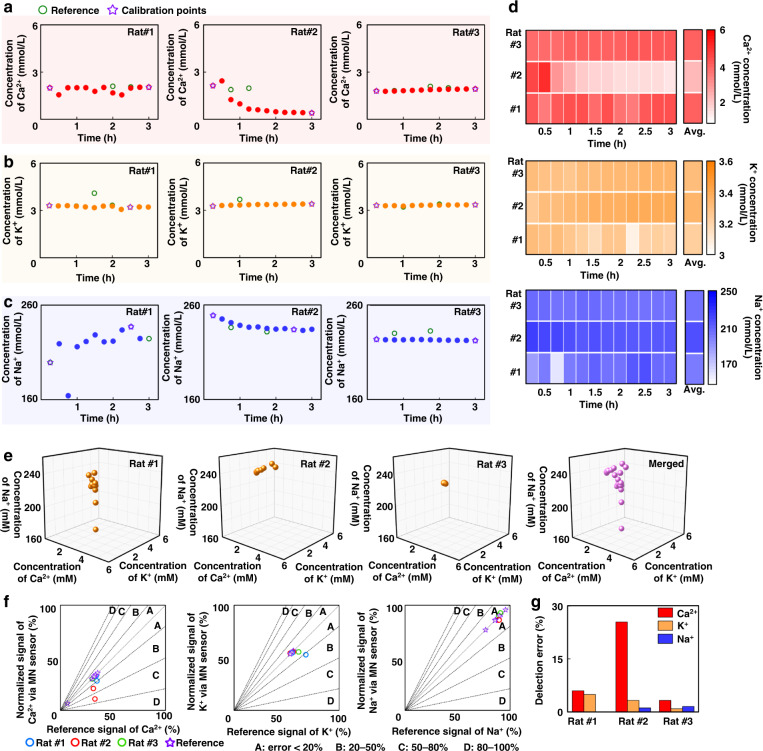
In vivo continuous ion monitoring of rats (*N* = 3) using an ion-sensing microneedle array for 3 h. **a**–**c** The ion-sensing microneedle array was pressed against the rats’ dorsal surface to penetrate the skin. The potentiometric signals detected by the ion-sensing microneedle array for each type of ion (Ca^2+^, K^+^, and Na^+^) were recorded. The potentiometric signals were converted into ion concentrations according to the standard curve. The reference concentrations of Ca^2+^, K^+^, and Na^+^ in the blood of rats were measured at certain time points using a commercial standard method, and the sensing results of the microneedle sensor were calibrated according to the reference concentrations (purple asterisks in the plots). By considering the difference between in vitro solution analysis and in vivo analysis, the calibration points were set at the first and last reference points for establishing the standard curves. **d** Heatmap plots summarizing the ion concentrations measured by the microneedle sensors. **e** Analysis of the correlations of the Ca^2+^, K^+^, and Na^+^ concentrations measured by the microneedle sensor in each rat. **f** Error grid analysis showing the detection accuracy of the microneedle sensor relative to reference concentrations while sensing different types of ions on each rat. The error range of each region is indicated in the figure. **g** In the in vivo experiment of each rat, the average error of the sensing result of the microneedle sensor relative to the reference value was calculated when different types of ions were detected

Next, we conducted in vivo experiments to verify the performance levels of the ion-sensing working electrodes in detecting fluctuations in subcutaneous Ca^2+^, K^+^, and Na^+^ in rats. Healthy rats were anesthetized, and the hair on their backs were removed. The ISMA was pressed onto the dorsal skin of the rats, allowing the microneedles to penetrate the stratum corneum. The voltage signals were then collected from the ion-sensing microneedle electrodes and measured every 15 min for a total of 3 h. In this study, we performed parallel experiments on three rats. By using the potentiometric signals collected from the ion-sensing working electrodes, the concentrations of Ca^2+^, K^+^, and Na^+^ were derived from the potential–ion concentration standard curves of the ion-sensing working electrodes. Blood samples from the tail arteries of the rats were collected at certain time points, and the ion concentrations in the blood were determined using a commercial biochemical analyzer as the reference. By considering the differences between the in vitro solution assays and in vivo assays, we calibrated the ion concentration results detected by the microneedle sensor to the reference results measured by a commercial biochemical analyzer at the first and the last time points. Figure 4a–c present the fluctuations in the Ca^2+^, K^+^, and Na^+^ concentrations detected by the microneedle sensor over time. The red, yellow, and blue curves correspond to the concentrations of Ca^2+^, K^+^, and Na^+^ measured by the microneedle sensor, respectively; the green dots in the figure show the reference ion concentration values obtained from the biochemical analyzer. After calibration with the reference ion concentration measured from blood, the microneedle sensor-recorded ion concentrations slightly fluctuated over time and were close to the blood ion concentrations. The microneedle sensor-recording results were summarized in the heatmap plot (Fig. 4d) to show the dynamic changes in ion concentrations during the recording period, demonstrating that the microneedle sensor was capable of continuously measuring the subcutaneous ion concentrations in rats.

To further analyze the ion fluctuations in the rats, the concentrations of Ca^2+^, K^+^, and Na^+^ measured by the microneedle sensor were statistically correlated for each rat. The correlations of the three types of ion concentrations appeared to vary for different rats, likely due to the physiological variations in individual rats (Fig. 4e). In addition, to verify the accuracy of the microneedle sensor relative to the actual concentration measured from blood, the sensing results via the microneedle sensor were analyzed and examined with error grid analysis (Fig. 4f); the average errors of the sensing results between the microneedle sensor and the reference were statistically quantified (Fig. 4g). In the error grid, most of the points were located in zones A and B, with values less than 20%; however, the sensing error of the Ca^2+^ concentration in rat#2 exceeded 20%, likely due to experimental noise. The results suggested that the microneedle sensor was capable of detecting Ca^2+^, K^+^, and Na^+^ concentrations with similar results to those detected by the biochemical analyzer. It should be noted that the high sensing accuracy was attributed to the use of data calibration with a two-point reference. The sensing results without calibration would generate significant error since the conversion of potentiometric signals into ion concentrations could be easily affected by the complicated in vivo environment. Nevertheless, these results indicated that the microneedle sensor could effectively monitor ion concentrations in situ in vivo, which could avoid the need for frequent sampling from blood.

### System integration of ISMA

**Fig. 5:**
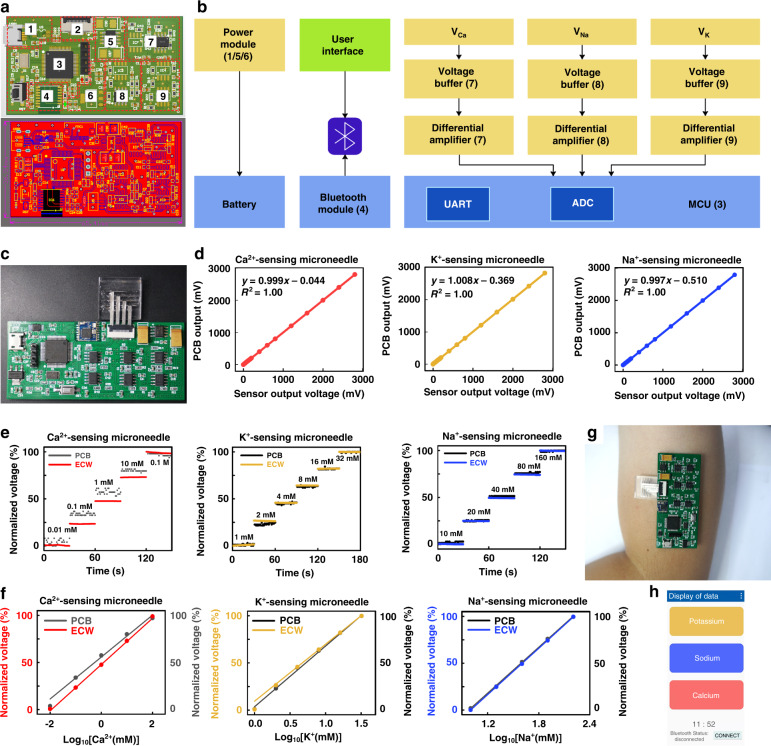
Design and functional verification of supporting circuit board for the ISMA. **a** Circuit implementation and layout design diagram of the printed circuit board. The red dashed boxes indicate the locations of the circuit components and modules. **b** System block diagram of the supporting circuit for ISMA. The circuit module contained the required integrated circuit chips and peripheral electronics for the potentiometric recording of three types of ions, signal processing, control and feedback, and Bluetooth wireless transmission circuitry. Thus, the module formed a compact system to support the functions of the microneedle device. The sensing circuit module consisted of three parallel sets of two-electrode systems. The two-electrode system enabled the recording of potentiometric signals that reflected the ion concentrations. **c** Photograph of the printed circuit board connected with the microneedle sensor. **d** Correlations of the input and output voltage signals from 0 V to 3000 mV by the printed circuit board; these correlations can be used for the potential detection of the concentrations of different ions. **e** Comparisons of the potentiometric responses of the ISMA to the respective analyte solutions in phosphate-buffered saline (PBS) using both a printed circuit board and electrochemical workstation (ECW). The detection range varies from 0.01 to 100 mM for Ca^2+^ sensing, from 1 to 32 mM for K^+^ detection and from 0.01 to 100 mM for Na^+^ sensing. **f** Comparisons of corresponding calibration plots of the ISMA to the different types of ions using both PCB and ECW. **g** Photographic image showing the prototype of ISMA with the size in accordance with the requirements to be worn on the arm or body. **h** Ion detection app interface formed through Bluetooth transmission in real time

We further developed a printed circuit board to support the functionalities of the ISMA. A conductive microneedle patch was designed with an extended lead that could be bent to the same plane as the circuit. This phenomenon enabled a compact connection between the assembled microneedle array and the supporting circuit. The ISMA was integrated with a printed circuit to form a demonstration system. Figure 5a, b depict the logic and pathways for signal conditioning, processing, and wireless transmission in the ISMA system, where the details of each labeled component are discussed in Figs. S5–S13 (Supplemental Material). The printed circuit boards were small enough (65.41 × 36.54 × 4.88 mm) to fit comfortably to the skin for potential wearable applications (Fig. 5c and Fig. S14, Supplemental Material).

The circuit module contained the required integrated circuit chips and peripheral electronics for the potentiometric recording of three types of ions, signal processing, control, feedback, and Bluetooth wireless transmission circuitry, thereby forming a compact system to support the functions of the microneedle device. The sensing circuit module consisted of three parallel sets of two-electrode systems. The two-electrode system enabled the recording of potentiometric signals that reflected the ion concentrations. The analog signals were transmitted to a microprogrammed control unit (MCU) using a digital-to-analog port in the microcontroller, and they were further converted into digital domains. The MCU outputs data through an onboard wireless transceiver for the real-time display of the recorded potentiometric signals. By using power from an external battery, the signals processed by the microcontroller were transmitted wirelessly via the Bluetooth module to a customized mobile application. The application consisted of a main interface (display of the levels of all physiological indicators) and subinterface (display of the trends of individual indicators) (Fig. S15, Supplemental Material). This phenomenon conceptually demonstrated that the circuit and app could potentially be used to support the operation of microneedle ion sensors.

As shown in Fig. 5d, the linear accuracies of the acquisition and output signals of the printed circuit board were characterized to maintain a stable signal transmission. The analog electrochemical signals for all three channels were precisely regulated, processed, and output (*R*^2^ = 1.00), indicating that the printed circuit board was reliably connected, stable in its signal acquisition and output, and usable for multichannel ion sensing. The performance of each ion-sensing electrode connected to the PCB was monitored separately with different analyte solutions; the performance levels were then compared with the results of the electrochemical workstation (ECW).

Figure 5e illustrates the potentiometric responses of ISMA in electrolyte solutions with physiologically relevant concentrations of 0.01 to 100 mM for Ca^2+^, 1–32 mM for K^+^ and 10–160 mM for Na^+^. By considering the difference in hardware impedance, the results recorded by both PCB and ECW were normalized to make a clearer comparison (absolute values are presented in Figs. S16 and S17, Supplemental Material). Linear relationships between potentiometric responses and analyte concentrations for three types of ions (Fig. 5f and Fig. S18, Supplemental Material) were observed, indicating that the signal detection on ISMA using PCB was consistent with that using ECW. The results shown above demonstrated that the ISMA had the potential to track changes in ion concentrations over a period and could respond rapidly when connected with a smartphone application as a wearable device. (Fig. 5g, h). The multi-ion-sensing microneedle array system supported by the miniature circuit design demonstrated excellent performance for specific-ion monitoring, suggesting its potential as advanced wearable electronics to detect human health states.

## Conclusion

In this study, we developed a multi-ion detection platform based on microneedle arrays that included ISMA, corresponding printed circuits, and mobile applications for the real-time monitoring of changes in Ca^2+^, K^+^, and Na^+^ concentrations to provide a quantitative diagnostic basis for the diagnosis and tracking of health states. ISMA was responsive to real-time changes in Ca^2+^, K^+^, and Na^+^ concentrations and exhibited the above physiological indicators with excellent linear response, selectivity, temporal stability, mechanical stability, reproducibility, and reliability characteristics regarding the signal transmission performance. ISMA continuously monitored multiple physiological signals that were conditioned, processed, and transmitted to smartphones in real time via a printed circuit with Bluetooth communication. The results from in vivo experiments showed that ISMA was capable of monitoring Ca^2+^, K^+^, and Na^+^ concentrations in real time and providing therapeutic recommendations, ultimately confirming the potential clinical applicability of ISMA. In this study, we provided a powerful strategy that could be widely used for ion detection in subcutaneous tissue fluids or even address other diseases.

## Methods

The following chemicals were obtained from Sigma‒Aldrich (USA): Na^+^-selective grade ionic carrier X, Na^+^ tetra [3,5-bis(trifluoromethyl)phenyl]borate (Na–TFPB), valinomycin (ionic carrier K^+^), Na^+^ tetraphenylborate (NaTPB), ETH 129 (Ca^2+^ carrier), 1-nitro-2-(n-octyloxy)benzene (NPOE), bis(2-ethylhexyl)sebacate (DOS), 3,4-ethylenedioxythiophene (EDOT), poly(4-styrenesulfonate) (NaPSS), HCl, H_2_SO_4_, urease (≥2 units/mg solid, from Candida spp.), bovine serum albumin (BSA), glutaraldehyde solution (20–25%), polyvinyl butyral resin BUTVAR B-98 (PVB), sodium chloride (NaCl), potassium chloride (KCl), calcium chloride (CaCl_2_), magnesium chloride (MgCl_2_), PBS (pH = 7.2), and methanol.

Au sulfite solution and Ag/AgCl ink were obtained from Yuncaitaotao Company. All the chemicals were used as received. All solutions were prepared using deionized water produced by Millipore Water Purification Systems, unless otherwise noted.

### Fabrication of microneedle electrode

A stainless steel (SUS304) material with a thickness of 200 µm was cleaned and dried using ethanol according to the design shown in Fig. 1b. A microneedle sheet with a conductive lead was cut using a laser machine, after which the lead of the microneedle sheet was bent at 90°, degreased, and washed. The oxidized layer on the surface was removed using acidic detergent, followed by the formation of a protective layer via electrochemical Au plating.

The laser used in the fabrication process was a YLP-F Series Optical Fiber Laser marking machine, with the following conditions: laser wavelength 1.06 μm, engraving line speed 2000 mm/s, power 16 W, and engraving 1000 times. The microneedles could be fabricated by Huasheng Precision Hardware Co., Ltd. The acidic detergent contained 24% zinc oxide, 30% ammonium chloride, 6% hydrochloric acid, 30% acetic acid, 12% deionized water, and 3% surfactant and was provided by the company Yuncaitaotao.

The conditions of gold plating were as follows: the modified electrode was used as the working electrode, the pure gold electrode was used as the counter electrode, and silver chloride was used as the reference electrode, forming a three-electrode system. The three electrodes were immersed in 0.2 mmol/L gold sulfite solution, and a constant voltage of −0.3 V was applied and maintained for 1200 s. A uniform layer of gold atoms was coated on the electrode surface. The thickness of the gold plating was not characterized in this work.

### Preparation of microneedle ion sensing electrodes

The sensing principle is demonstrated in Fig. S19 (Supplemental Material). First, a Na^+^-selective membrane mixture consisting of Na^+^ ionophore X (1 wt.%), Na–TFPB (0.55 wt.%), PVC (33 wt.%), and DOS (65.45 wt.%) was prepared. The mixture (200 mg) was dissolved in 1320 μL tetrahydrofuran and shaken for 30 min on a shaker. The mixture for the K^+^-selective membranes consisted of valinomycin (2 wt.%), NaTPB (0.5 wt.%), PVC (32.7 wt.%), and DOS (64.7 wt.%). The membrane-modification solution was obtained by dissolving the cocktail (200 mg) in 700 μL cyclohexanone. Similarly, the Ca^2+^-selective membrane mixture consisted of ETH 129 (0.46 wt.%), NaTPB (0.48 wt.%), PVC (33.02 wt.%), and NPOE (66.04 wt.%). Membrane-modification solutions were prepared by dissolving 200 mg of the mixture in 1 mL of tetrahydrofuran. All ion-selective solutions were stored in a sealed container at 4 °C until use.

To minimize the potential drift at the ion-selective electrode (ISE), we used poly(3,4-ethylenedioxythiophene) polystyrene sulfonate (PEDOT:PSS) as the ion-to-electron sensor and deposited it on the working electrode. Specifically, electrodes were prepared via constant-current electrochemical polymerization using a solution of 0.01 M PEDOT and 0.1 M NaPSS. A constant current of 14 μA was applied to each electrode for 720 s. Subsequently, ion-selective membranes were prepared by dripping 10 μL Na^+^-selective membrane mixture, 4 μL K^+^-selective membrane mixture, and 5 μL Ca^2+^-selective membrane mixture onto the corresponding electrodes. The electrodes were allowed to dry overnight in an ambient environment and were then ready for testing.

### Fabrication of Ag/AgCl reference electrode

A piece of laser-cut microneedle sheet was placed in a stainless steel flux and ultrasonically soaked for 5 h. The sheet was then washed with 75% alcohol, wiped dry, and plated in a Au sulfite solution for chemical deposition at −20 mA for 1200 s. After ultrasonic soaking in 75% alcohol for 5 min, the tip was dried, uniformly coated with Ag/AgCl ink, and dried at 90 °C for 1 h. The prepared reference electrode was then dipped in a 5% (wt.%) PVB solution using the immersion coating method and dried at room temperature for 12 h.

### Prototype assembly

For each patch of the ion-sensing microneedle electrode and Ag/AgCl reference electrode, the conductive lead on the back of the microneedle electrode was physically bent at 90°. The electrodes were then arranged in parallel at 3 mmol/L intervals, combined using an acrylic plate designed by AutoCAD (Autodesk, USA), engraved, and processed using a laser to form the ion-sensing microneedle array. The bent part of the conductive lead was then cast and insulated using PDMS, after which the ends of the leads were trimmed uniformly and easily connected to the PCB interface to integrate the prototype.

### Fabrication of the printed circuit board

The printed circuit board was designed and wired by using the AutoCAD designer, and it was printed and aligned for soldering. After connecting the electrodes through the sensor interface, the board was powered by a battery module to collect potentiometric signals from the surface of the ion-sensing microneedle electrode. The signals were processed through a two-stage differential circuit to reduce signal distortion and common-mode noise interference. Subsequently, the collected analog signal was converted into a digital signal using the STM32 chip through analog-to-digital conversion, and a gradient conversion was performed according to the calibration curve obtained from in vitro experiments to obtain the corresponding ion concentration. The concentration was transmitted to the Bluetooth module via a serial port to send the concentration data of different ions to the mobile terminal that displays the trend graph of ion changes through the interface that was designed using LabVIEW software (NI, USA).

### Fabrication of ISMA prototype

The leads of the modified ion-sensing microneedle electrodes were bent by 90° to form an L-shaped microneedle electrode; suitable holes were cut into an acrylic plate with a thickness of 3 mm using a laser cutter. The microneedle electrodes were then embedded parallel to the holes to form a side-by-side microneedle sensing array. Subsequently, the leads of the ion microneedle electrodes were covered and insulated with PDMS to prevent short-circuit accidents. The excess conductive leads of the microneedle electrode were removed with pliers to produce a uniform and flat cut of the microneedle electrode leads for a stable and convenient connection to the sensor interface.

### SEM characterization

We used a scanning electron microscope to characterize the surface morphologies of the prepared microneedle sheets and the modified microneedle electrodes. It could be clearly observed from the figure that the surfaces of the blank microneedles were flat without obvious defects. The surfaces of the microneedles deposited with Au were uniform and flush with no observable bumps on the surface. After modifying the surface of the Au electrode with PEDOT, it was observed that the surface had a lamellar-like structure with small particles on the edges. After modifying the Na^+^-selective membrane, clear hillock-like protrusions appeared on the surface of the microneedle electrode and on some particles at the edge. After modifying the K^+^-selective membrane, the surface of the microneedle electrode was relatively flat; however, clear lines were observed, and there were no observable particles at the edges. After modifying the Ca^2+^-selective membrane, the surface of the microneedle electrode was relatively flat; however, clear lines were observed, and there were no observable particles at the edges.

### Fluorescence characterization

An aqueous solution of rhodamine B was prepared at a concentration of 2 mg/mL, and a cotton swab was used to collect a small amount of the solution and drop it on the head of the microneedle to coat the stain evenly on the surface of the microneedle tip. A piece of fresh porcine skin was utilized as a substitute for human skin. After the grease on the surface of the skin was absorbed using blotting paper, the skin was cleaned and placed flat on the foam. A microneedle tip was placed perpendicular to the surface of the porcine skin and inserted into the surface of the porcine skin with force. The specimen was left to stand for 3 min before being removed. The porcine skin was observed after the microneedle transdermal treatment using an optical microscope. To demonstrate the transdermal performance of the microneedle tip, a thin slice of the microneedle transdermal-treated porcine skin with a thickness of approximately 200 μm was dissected along the cross section of the hole left by the microneedle transdermal using a razor blade; it was placed under a fluorescence microscope for observation with red fluorescence.

### Microneedle electrode in vitro sensing characterization

A 25 mL beaker containing the solution was utilized, and a twice-folded cling film that was used to simulate the skin was fixed onto the surface of the beaker. An ion-sensing microneedle array was used to puncture the cling film to simulate skin penetration, and it was fully exposed to the solution to detect ions in the solution. A series of characterizations of the electrochemical sensors was performed using a CHI 760E workstation (CH Instruments Inc., USA) and commercial Ag/AgCl electrodes to evaluate the performance levels of the working electrodes. A two-electrode system was constructed to test the performance of the ion sensor in deionized water.

Step-response tests were performed on all ion sensors using an ion microneedle electrode as the working electrode and a commercial Ag/AgCl electrode as the counter electrode to evaluate their linear response capability for each subject over a specific range. During the test, each change in the solution required a test pause of 60 s to allow the complete diffusion of the solute in the solution. For the detection of Ca^2+^, the concentration of Ca^2+^ in the solution increased gradually from 0.01 to 0.1, 1, 10, and 100 mmol/L, and the response voltage of the electrode gradually increased with the change in the concentration of Ca^2+^. Similarly, to detect K^+^, the concentration of K^+^ in the solution gradually increased from 1 to 2, 4, 8, 16, and 32 mmol/L; the response signal of the electrode to the ions gradually increased with an increase in the K^+^ concentration. Similarly, to detect Na^+^, the concentration of Na^+^ in the solution gradually changed from 5 to 10, 20, 40, 80, and 160 mmol/L. The response signal of the electrode gradually increased as the concentration of Na^+^ increased.

### Stability, reproducibility, and selectivity tests of microneedle electrodes

To determine the stabilities of the ion sensors, we tested the same sensors with five sets of signals at three fixed concentration steps. We investigated the temporal stabilities of the ion sensors by performing sensitivity tests three times a day for one week after their preparation, and the daily results were averaged. The ion sensors were stored at room temperature in a light-proof environment. To obtain more reliable results, all the electrochemical sensors were subjected to concentration changes during the measurement period in the concentration range of the step response test.

The reproducibility of each ion was assessed by testing the slopes and intercepts of the five microneedle ion electrodes. As shown in Fig. 3g, the average slope and intercept values for the Na^+^ channel were 50.7 mV/decade and 221.6 mV, respectively, with relative standard deviations (RSDs) of 2.3 and 2.4%, respectively. The measured slope and intercept values for the K^+^ channel were 53.6 mV/decade with an RSD of 2.5% and 236.8 mV with an RSD of 2.2%, respectively; these results indicated satisfactory reproducibility of the microneedle ion electrode between batches to detect individual ions. Thus, the prepared ion-sensing microneedle electrode exhibited high stability and met the requirements for home smart sensing.

For the selective detection of different ions, we constructed a two-electrode system to test each microneedle ion sensor. The system was evaluated by sequentially adding markers and multiple interfering substances at a particular concentration for the corresponding markers. Specifically, for the Na^+^ detection electrode, K^+^, Mg^2+^, Ca^2+^, and Na^+^ were added sequentially to the 10 mmol/L NaCl solution (the concentration of each ion corresponds to the proportion of the actual concentration of the test marker), and the change in the voltage signal was obtained. For the Ca^2+^ detection electrode, Na^+^, Mg^2+^, K^+^, and Ca^2+^ were sequentially added to a 0.5 mmol/L CaCl_2_ solution (the concentration of each ion corresponds to the proportion of the actual concentration of the test specimen), and the voltage signal was obtained. Similarly, for the K^+^ detection electrode, Na^+^, Mg^2+^, Ca^2+^, and K^+^ were sequentially added to a 2 mmol/L K^+^ solution (the concentration of each ion corresponds to the proportion of the actual concentration of the test specimen), and the changes in the voltage signal were obtained. The relative signal shift was evaluated using the change in voltage signal before and after marker addition and by setting the change in the absolute signal generated by each electrochemical sensor before and after the marker concentration increased to 100%. Thus, the signal difference between each interfering substance addition and the initial concentration was normalized.

### In vivo experiments

In vivo experiments were conducted using rats to better evaluate the role of the microneedle sensor in animal monitoring. The study was approved by the Institutional Animal Care and Use Committee of Sun Yat-Sen University. All animals received humane care in accordance with institutional guidelines. C57BL/6 rats were obtained from the Sun Yat-Sen University Animal Facility and used for experimental studies.

First, gas anesthesia was applied to the rats, and their backs were depilated using surgical scissors and depilatory cream to obtain bare skin with an area of 3 × 4 cm^2^. Subsequently, the microneedle sensor was applied over the depilatory area, and it penetrated the skin of the rats. This process allowed the microneedle-array electrode to fit with the skin well and to penetrate the stratum corneum. The voltage signal of the ion-sensing microneedle electrode was collected, and measurements were acquired every 15 min for 3 h. In this study, we performed parallel experiments on three rats. The standard voltage-ion concentration curves of the microneedle electrodes were used to convert the signals collected from the microneedle Na^+^, K^+^, and Ca^2+^ sensors to concentrations of different types of ions. Blood was collected from the tail arteries of rats at certain time points; a biochemical analyzer was used to determine the ion concentration in the blood as a reference value. The ion concentrations measured at the first and last time points were used to calibrate the differences between the in vitro solution assays and in vivo assays. During the in vivo experiment, microneedles penetrated through the skin, but no bleeding was observed. Furthermore, the lengths of microneedles could be flexibly tuned by integrating a porous spacer (with a certain thickness), such as a polyimide or polyethylene glycol terephthalate substrate (as shown in Fig. S20, Supplemental Material). and the length of the microneedle penetrating the skin could be effectively regulated.

## Supplementary information

Supplemental material-revised version
