# Supplementary material for: 3D-assembled microneedle ion sensor-based wearable system for the transdermal monitoring of physiological ion fluctuations
Source: Microsyst Nanoeng. 2023 Mar 9;9:25. doi: 10.1038/s41378-023-00497-0 (PMC9998623; doi:10.1038/s41378-023-00497-0)
Supplement: Supplementary file 1 — Supplemental material-revised version [file 41378_2023_497_MOESM1_ESM.docx]

**Supplemental Material**

**3D-assembled microneedle ion sensor-based wearable system for transdermal monitoring of physiological ion fluctuations**

Xinshuo Huang,^1,+^ Shantao Zheng,^1,+^ Baoming Liang,^1^ Mengyi He,^1^ Feifei Wu, ^1,3^ Jingbo Yang,^2^ Hui-jiuan Chen,^1,^* Xi Xie^1,2,^*

^1^State Key Laboratory of Optoelectronic Materials and Technologies, School of Electronics and Information Technology; Guangdong Province Key Laboratory of Display Material and Technology, Sun Yat-Sen University, Guangzhou, China

^2^School of Biomedical Engineering, Sun Yat-Sen University, Guangzhou, China

^3^Pazhou Lab, Guangzhou 510330, China

* Corresponding authors, E-mail: xiexi27@mail.sysu.edu.cn

+These authors contributed equally to this work.


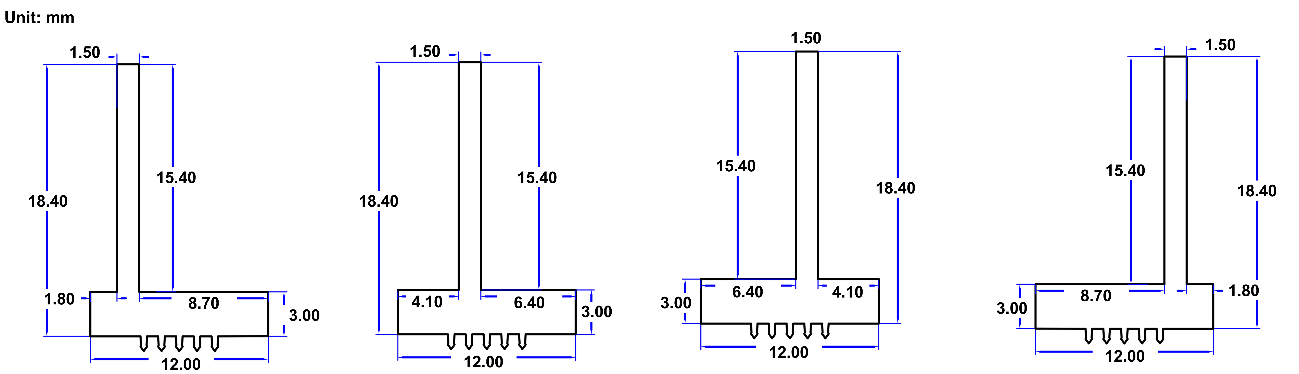


**Figure S1.** The photograph showing the design of the microneedle electrode by AutoCAD. Each electrode processed 5 needles with the length of about 800 μm and a form back for the fabrication of the microneedle array, together with a lead up to 15 mm which was suitable for bent and connection with the printed circuit board for data transmission.


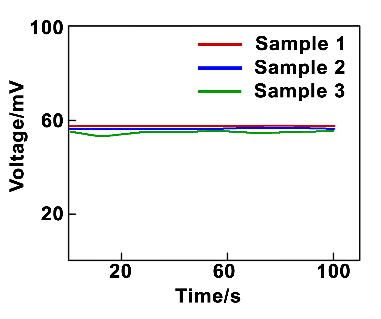


**Figure S2.** Graph showing the characterization of stability of Ag/AgCl reference electrode. Results showed above revealed the stability of Ag/AgCl reference electrode with a voltage drift of around 50 mV.


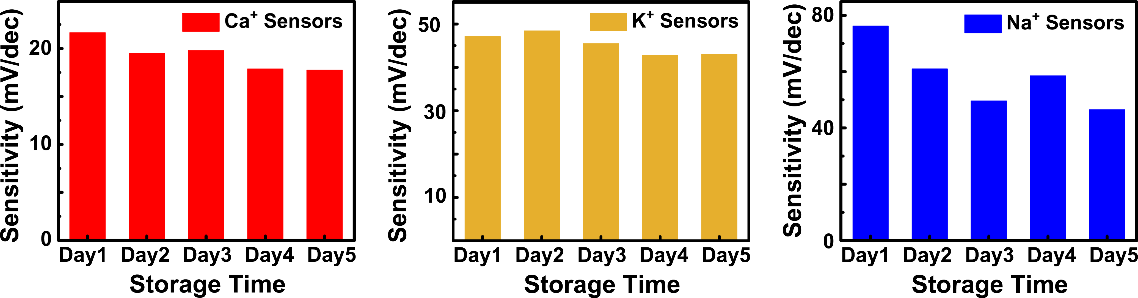


**Figure S3.** Graph showing the characterization of reproductivity of ion-sensing microneedle electrodes in a week. Daily sensitivity changes for each electrochemical sensor over a week was estimated. Results suggested the Ca^2+^ and K^+^ sensing electrode maintained excellent temporal stability while the Na^+^ sensing electrodes showed a significant change that are hard to ignore in sensitivity from day 2 onwards. As shown above, the final relative sensitivities of these sensors after 4 days were 61.00% for the Na^+^ sensing electrode, 91.25% for the K^+^ sensing electrode, and 81.98% for the Ca^2+^ sensing electrode. The results above revealed that the sensitivity of the ion-sensing microneedle electrodes remained stable over 4 days of storage, showing the importance of pre-calibration in improving the stability of the sensors.


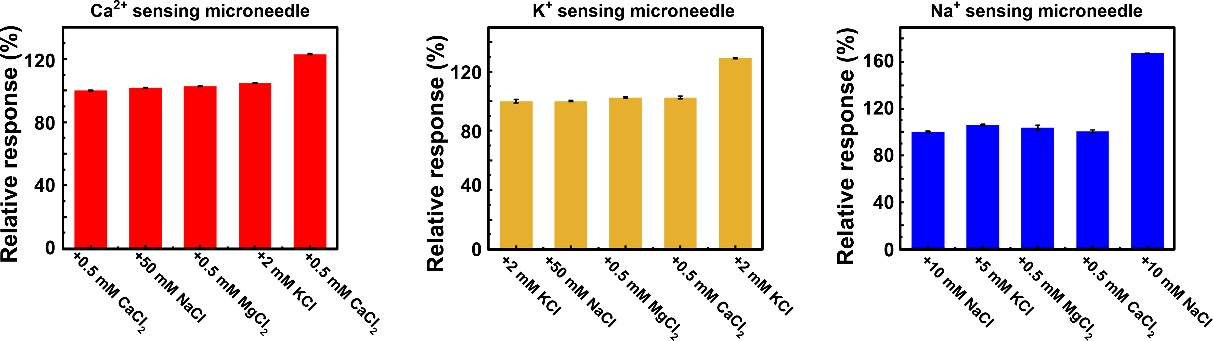


**Figure S4.** Graph showing the selectivity of ion-sensing microneedle electrodes for the sensing of Ca^2+^, K^+^ and Na^+^ (relative value). We tested the selectivity of electrode detection by sequentially adding Na^+^, Mg^2+^ and K^+^ ions to the test solution for microneedle Ca^2+^ electrode. For a better discussion, we viewed the sensing signal of the first time as 100%, with the signals followed converted into relative values. The results showed that the microneedle Ca^2+^ electrode showed a significant voltage signal (>20 %) only for the presence of Ca^2+^, while the presence of Na^+^, Mg^2+^, K^+^ produced only a weak voltage signal (<2 %) for the electrode, demonstrating that the microneedle Ca^2+^ electrode can selectively detect K^+^ and avoid the interference of other ions. These results demonstrated that the prepared microneedle ion electrode was able to sensitively and selectively detect the concentration changes of the target ions with a good linear range consistent with the application of in vivo detection.


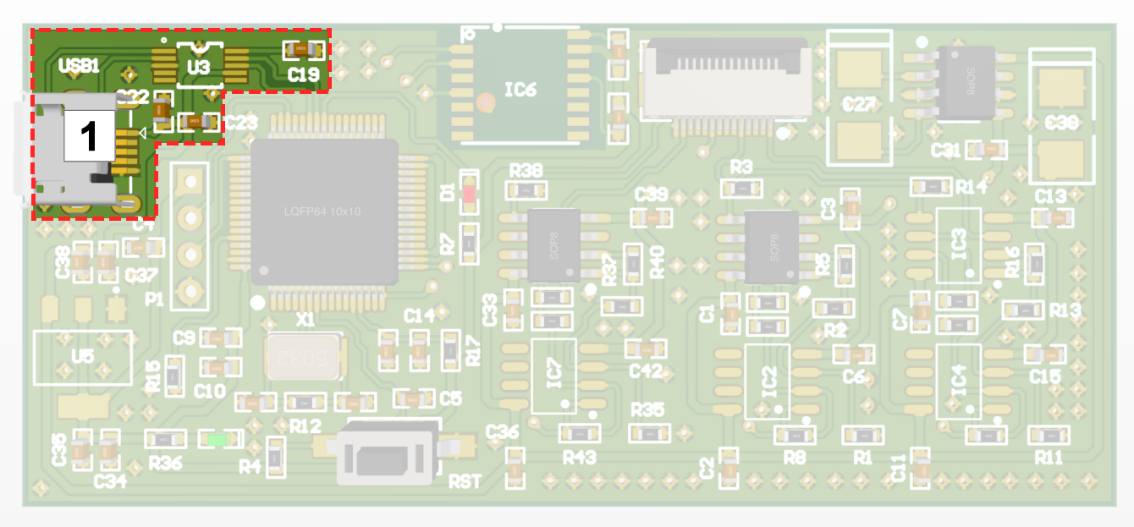


**Figure S5.** Circuit diagram showing the Micro-USB serial port (Circuit Component #1) of ISMA. The serial port provided power to the board which also allow for data transfer.


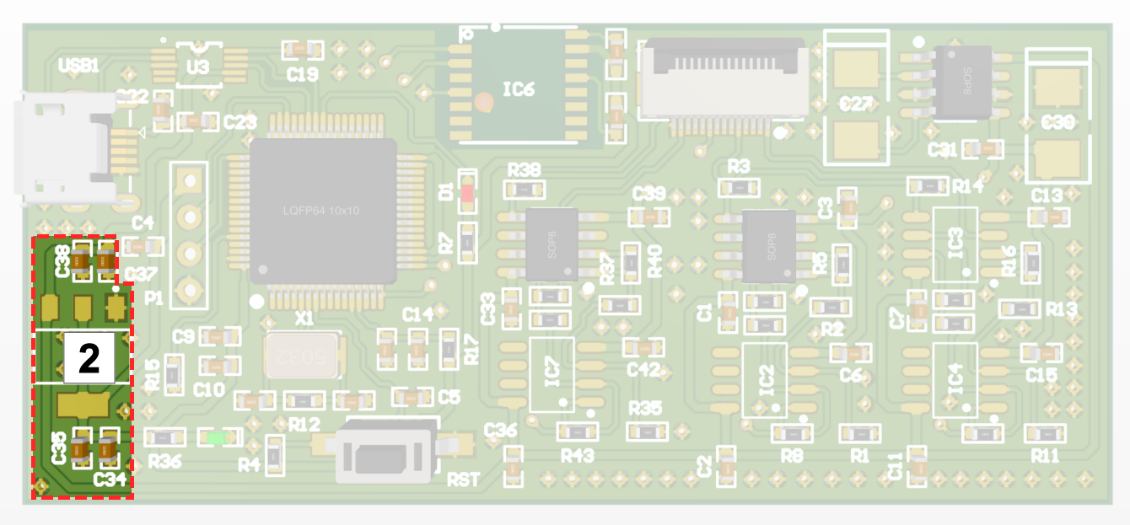


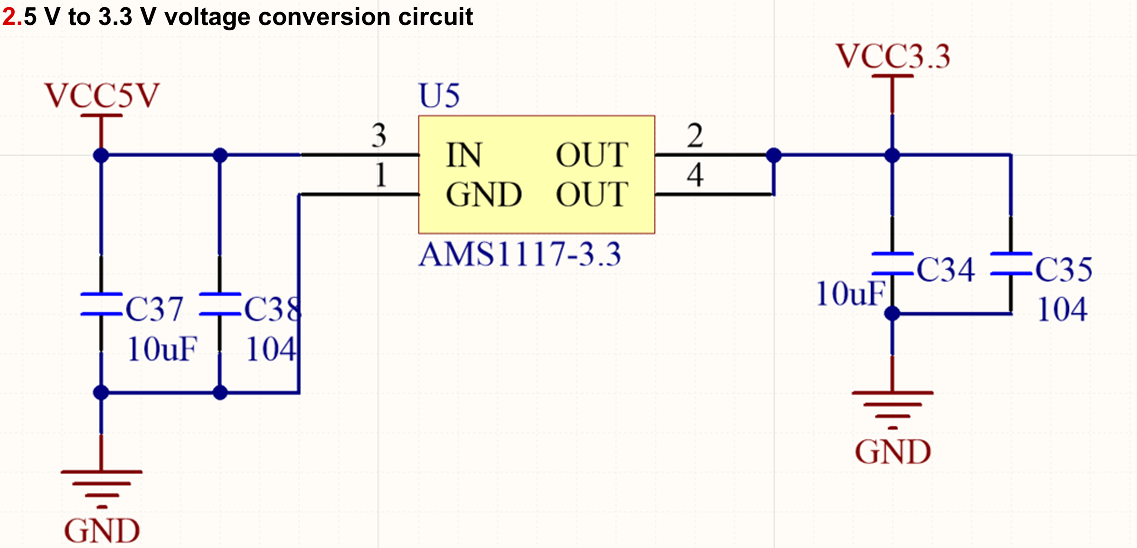


**Figure S6.** Circuit diagram showing the 5 V to 3.3 V voltage conversion circuit (Circuit Component #2) of ISMA. This module was used for 3.3V voltage generation. The voltage conversion circuit transferred 5 V to 3.3 V by the voltage regulator AMS1117-3.3. The converter output 3.3 V voltage at pin 2. The 3.3 V-voltage provided power support for the STM32 minimum single-chip system and the Bluetooth section.


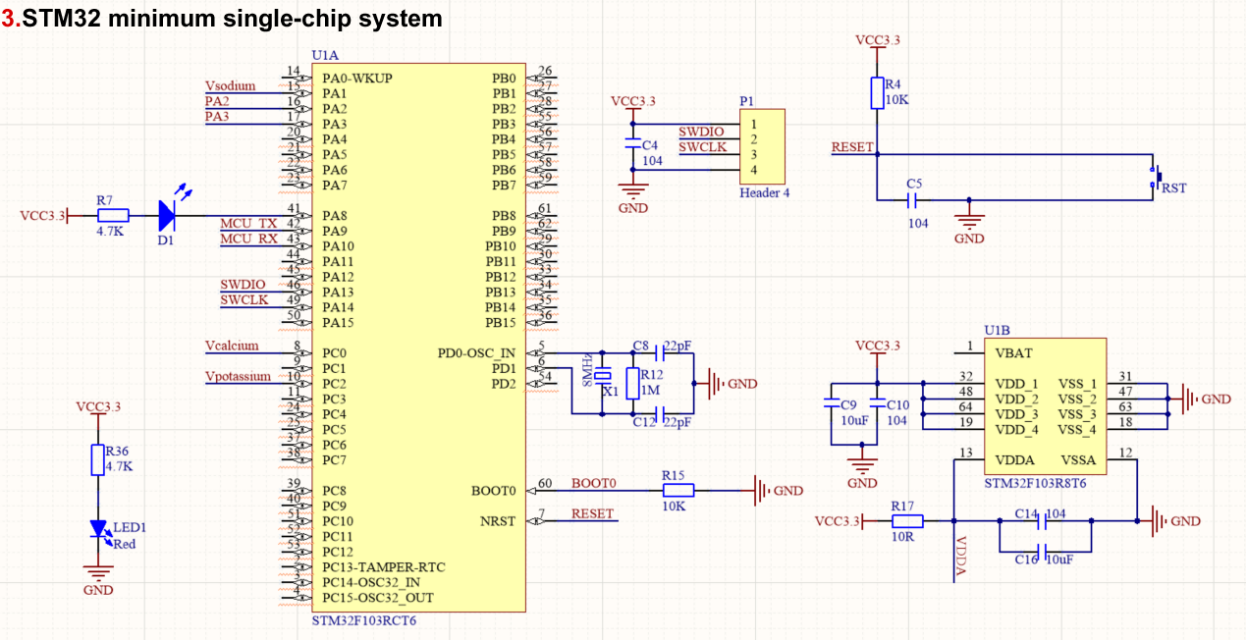

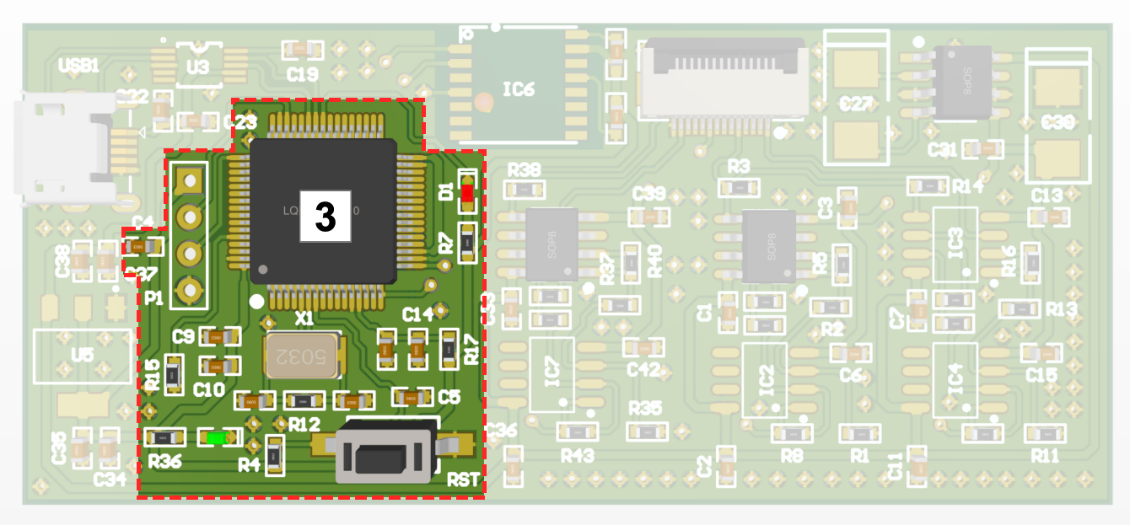


**Figure S7.** Circuit diagram showing the STM32 minimum single-chip system (Circuit Component #3) of ISMA. The STM32 minimum single-chip system included the following modules: 1. st-link interface, which can burn the program to the chip and power the PCB board. 2. clock module, which obtains the chip's clock signal through an external 8MHz crystal. 3. circuit reset module, which uses a key switch to reset the STM32 chip's running program. 4. data processing part, which converts the analog signal obtained from the sensor and detection circuit into a digital signal for corresponding processing and sends the data to the mobile terminal through the serial port to the Bluetooth module.


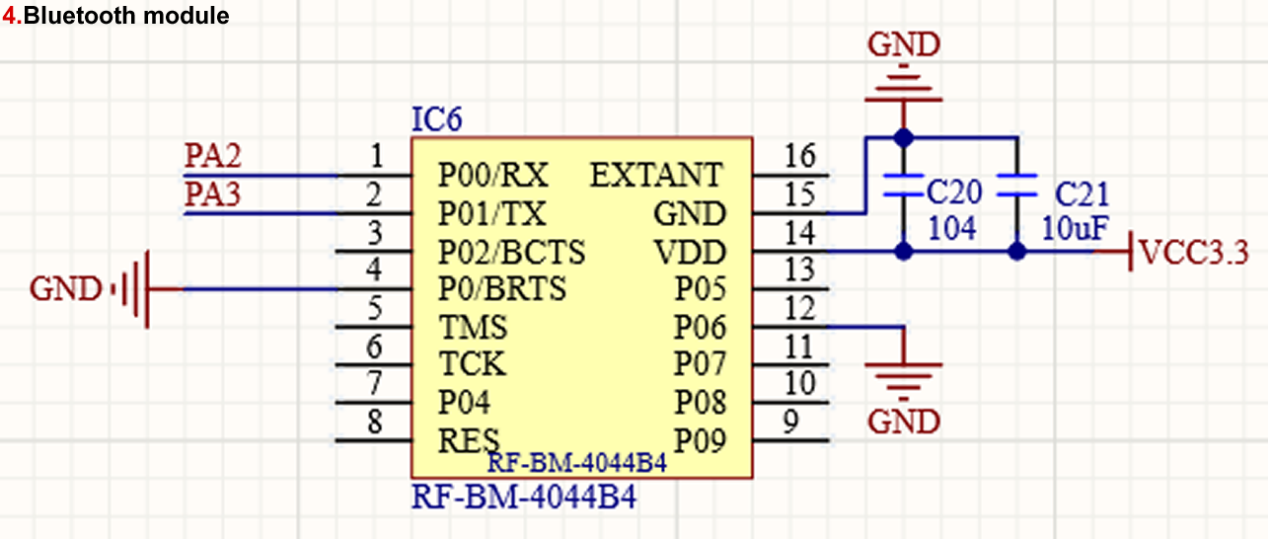

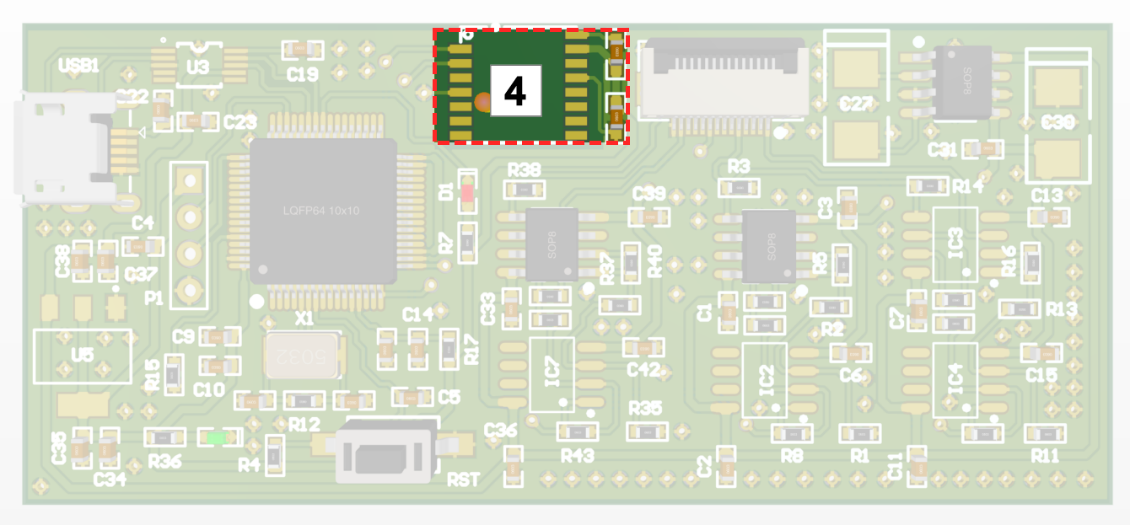


**Figure S8.** Circuit diagram showing the Bluetooth module (Circuit Component #4) of ISMA. The low-power Bluetooth module was connected to the STM32 minimum single-chip system through the serial port, and sent data to the smart phone terminal by wireless transmission, where the data was displayed and saved.


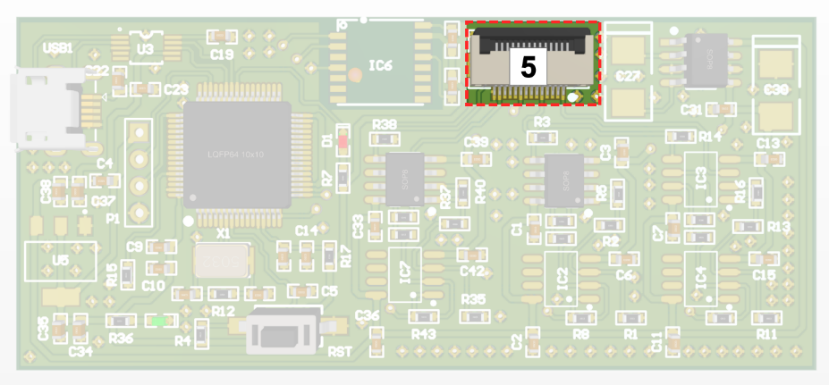

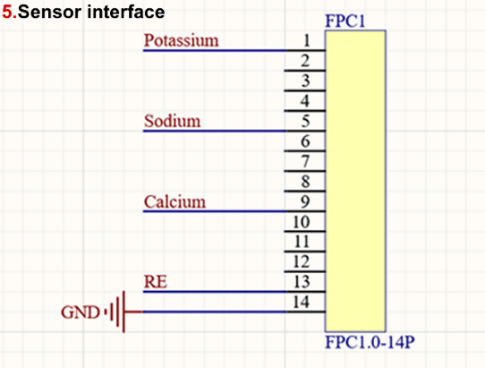


**Figure S9.** Circuit diagram showing the sensor interface (Circuit Component #5) of ISMA. The prepared microneedle sensor was connected to the PCB board through this interface and allow the transmission of the signal detected to the PCB board.


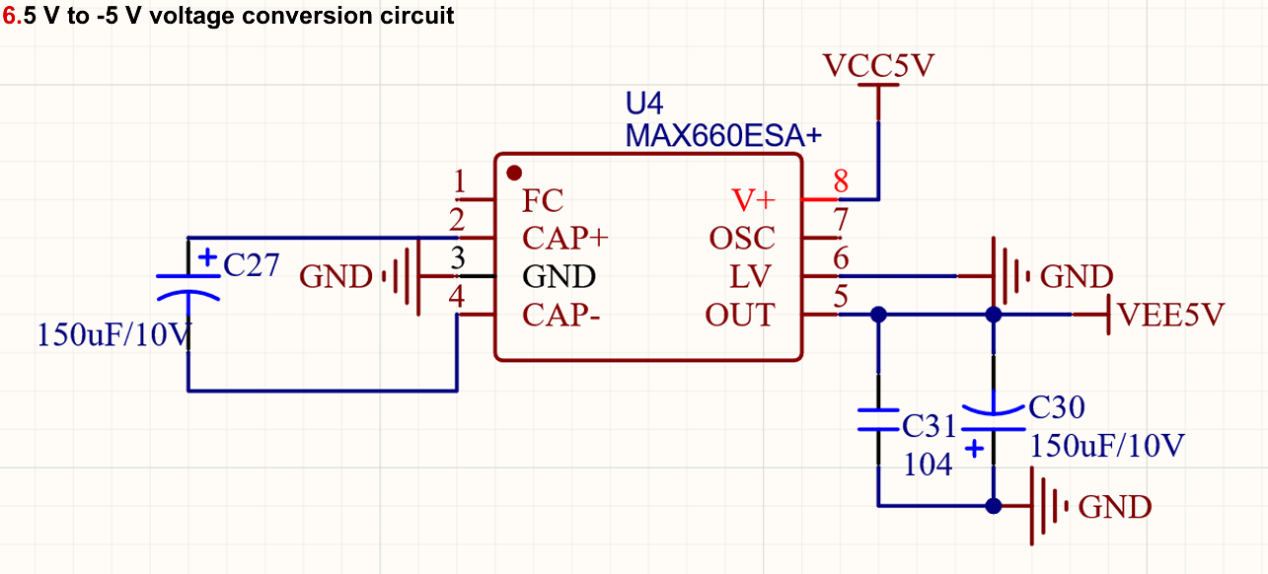

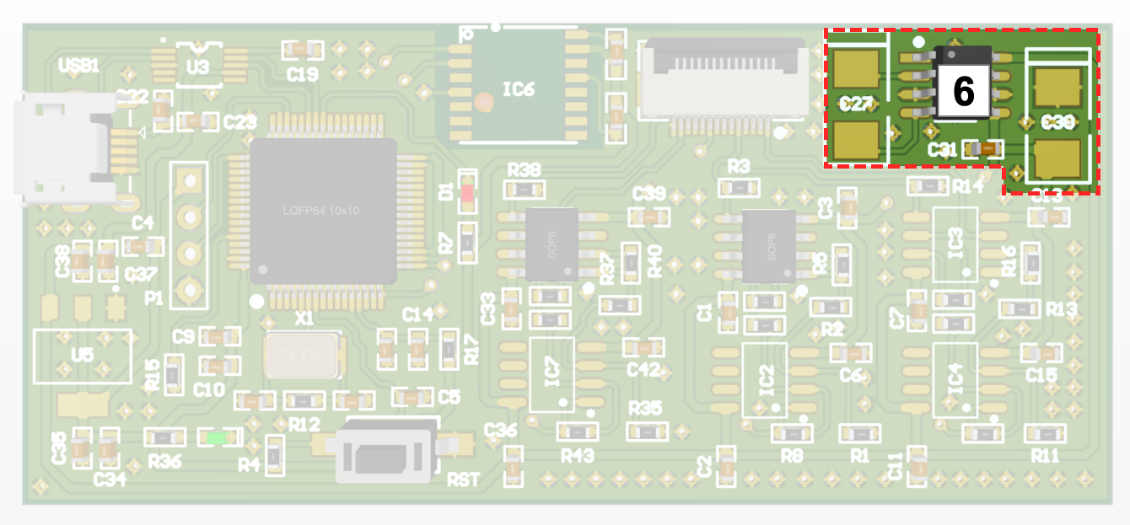


**Figure S10.** Circuit diagram showing the 5 V to -5 V voltage conversion circuit (Circuit Component #6) of ISMA. This module generated a -5V voltage from the MAX660ESA+ chip to power the operational amplifier in the ion detection circuit module. The converter output -5 V voltage at pin5.


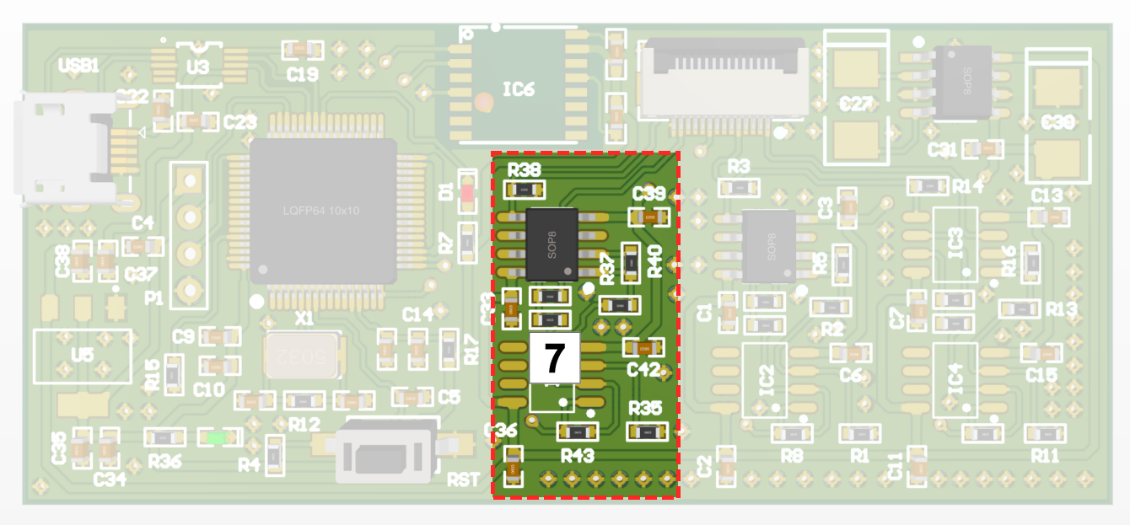


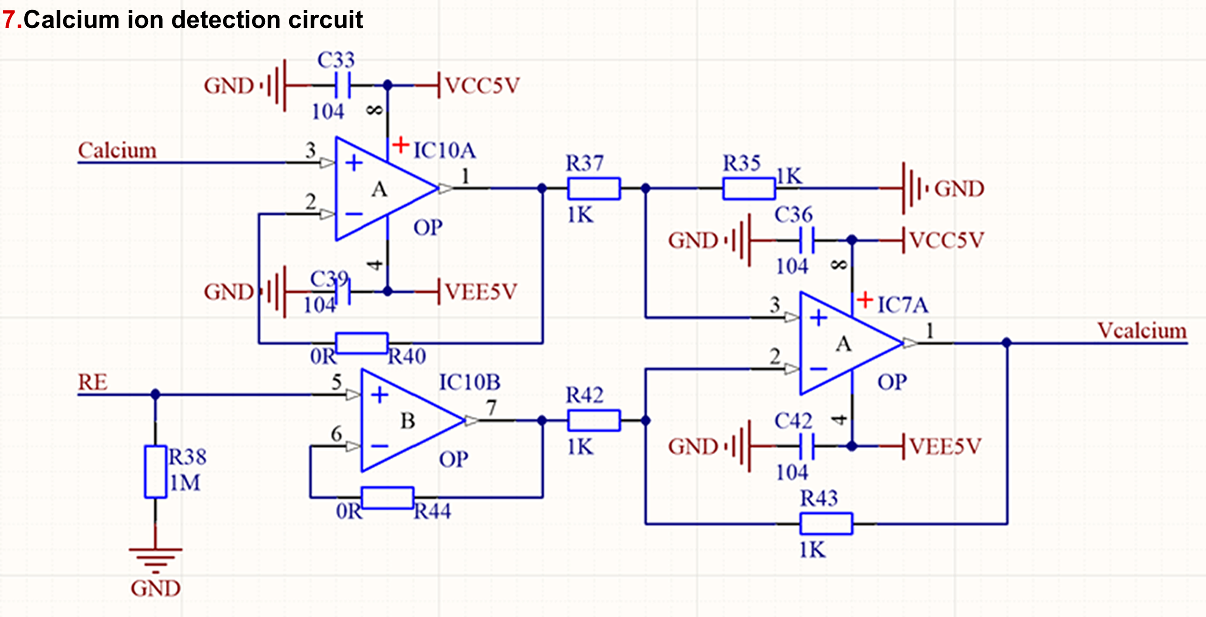


**Figure S11.** Circuit diagram showing the Ca^2+^ detection circuit (Circuit Component #7) of ISMA. The signal obtained from the voltage-based Ca^2+^ electrode was processed by a two-stage differential circuit. The first stage was an in-phase differential input used as a voltage follower, which could separate the two stages, enhance the carrying capacity, reduce the impact on the latter circuit, and the higher input impedance could reduce the distortion of the input signal. The second stage was a differential amplifier circuit, which could reduce the interference of common mode noise and output the Ca^2+^ voltage signal between the two electrodes more accurately.


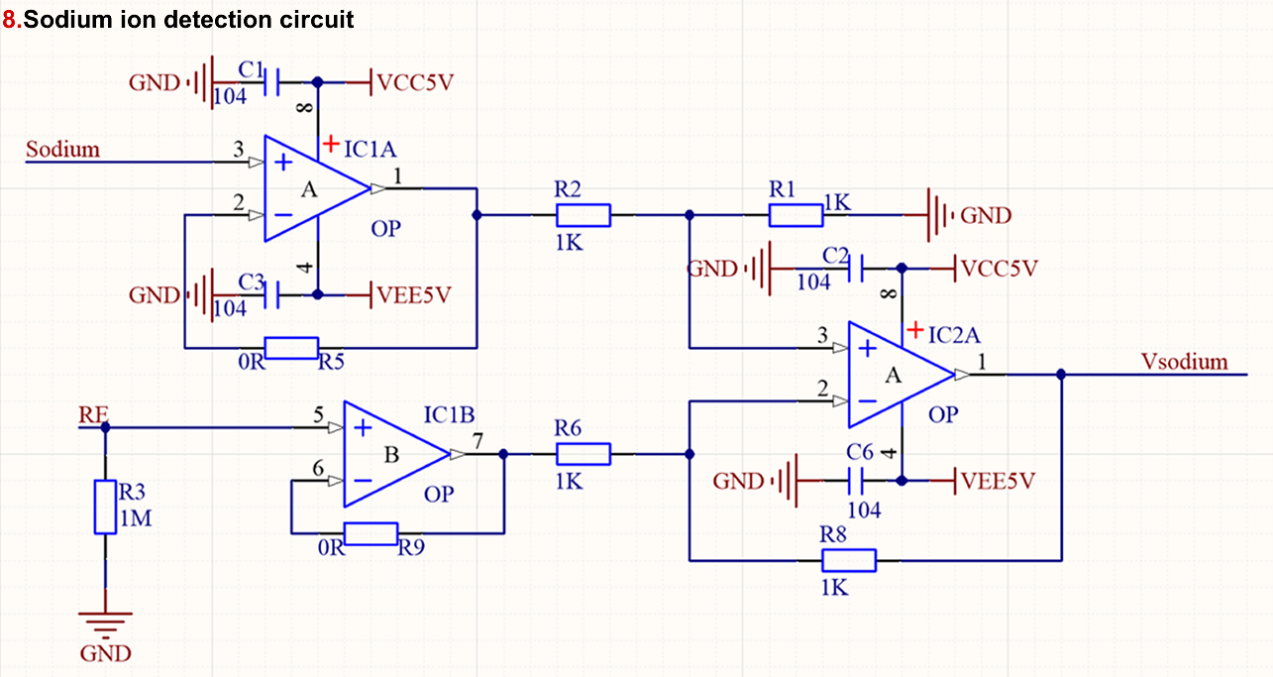

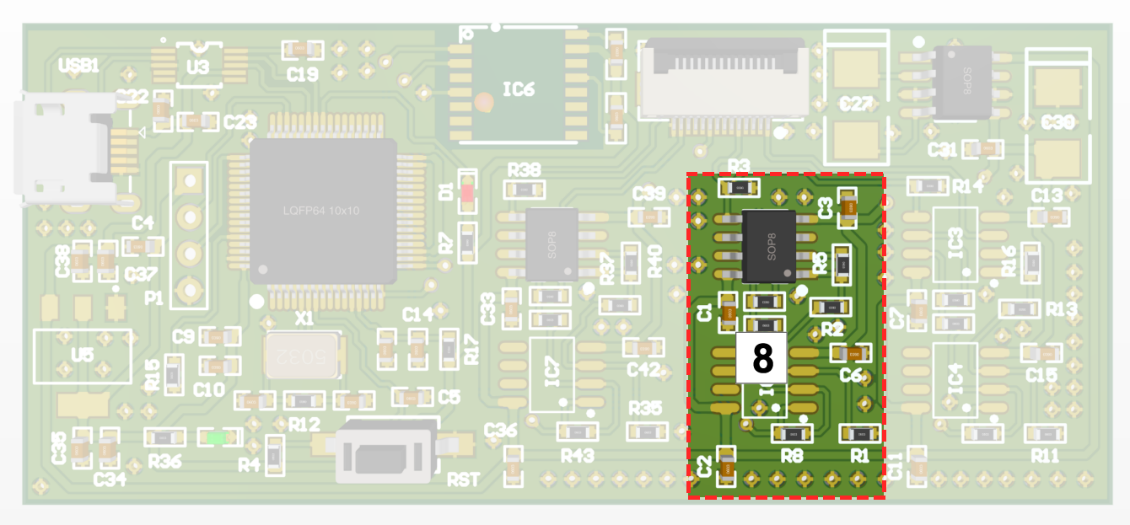


**Figure S12.** Circuit diagram showing the Na^+^ detection circuit (Circuit Component #8) of ISMA. The signal obtained from the voltage-based Na^+^ electrode was processed by a two-stage differential circuit. The first stage was an in-phase differential input used as a voltage follower, which could separate the two stages, enhance the carrying capacity, reduce the impact on the latter circuit, and the higher input impedance could reduce the distortion of the input signal. The second stage was a differential amplifier circuit, which can reduce the interference of common mode noise and output the Na^+^ voltage signal between the two electrodes more accurately.


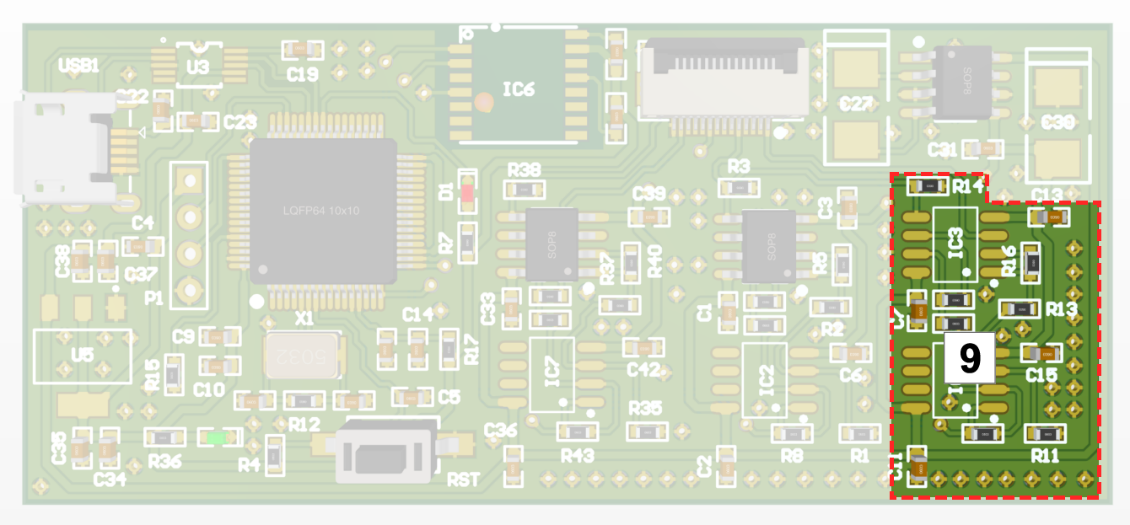


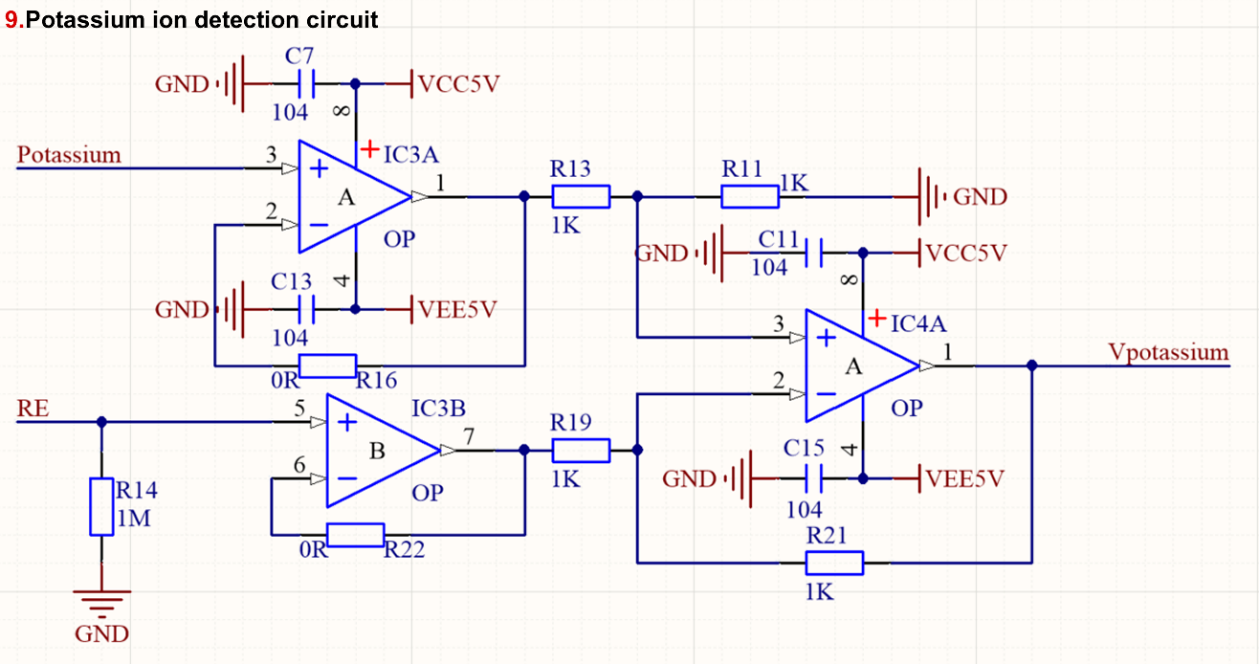


**Figure S13.** Circuit diagram showing the K^+^ detection circuit (Circuit Component #9) of ISMA. The signal obtained from the voltage-based K^+^ sensor was processed by a two-stage differential circuit. The first stage was an in-phase differential input used as a voltage follower, which separated the two stages to enhance the carrying capacity and reduced the impact on the later stages, and the higher input impedance reduces the distortion of the input signal. The second stage was a differential amplifier circuit, which could reduce the interference of common mode noise and output the K^+^ voltage signal between the two electrodes more accurately.


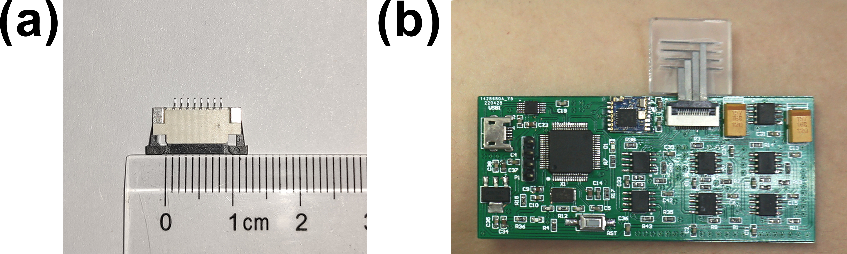


**Figure S14.** The photograph showing the characterization of sensor interface and printed circuit board. a. The sensor interface was designed and provided by Yuncaitaotao Company with the size of 17 mm in length. b. The printed circuit board connected with the prototype through the sensor interface.


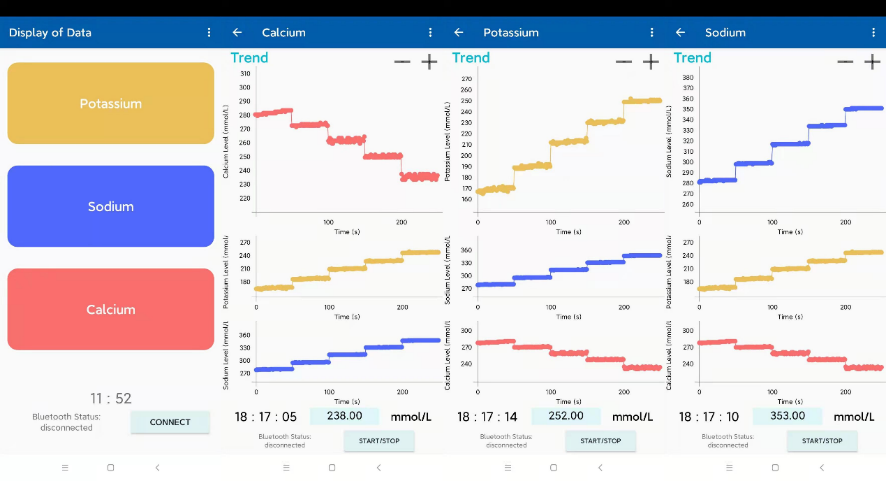


**Figure S15.** The photograph showing the detail interface of the application design on smartphone. The APP could be further developed. Functions of signal calculation into concentration of Ca^2+^, K^+^, and Na^+^ were showed above.


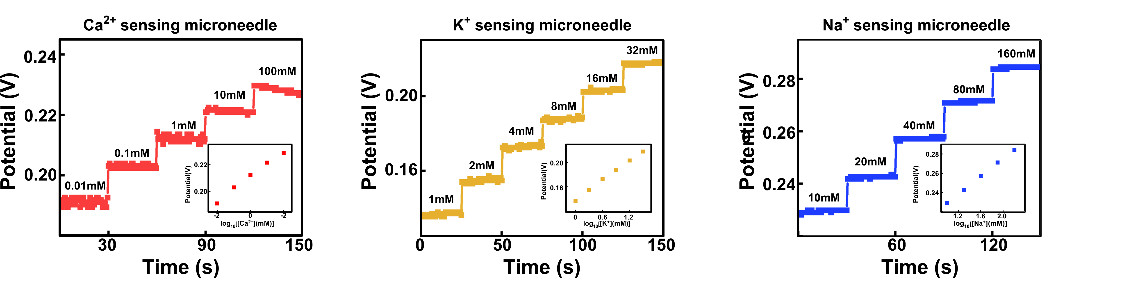


**Figure S16.** Graph showing the open-circuit potential responses of the Ca^2+^, K^+^ and Na^+^ collected with the ion-sensing microneedle electrodes connected with printed circuit board, respectively. Figures above showed the potential signals and time using ISMA with as-prepared PCB.

The potentiometric signal of the microneedle possessed good linearity with the Ca^2+^ concentration, with a detection sensitivity of 8.79 mV (R^2^= 0.9869) per decade of concentration.

The potentiometric signal of the microneedle possessed good linearity with the K^+^ concentration, with a detection sensitivity of 52.9 mV (R^2^= 0.9977) per decade of concentration.

The potentiometric signal of the microneedle possessed good linearity with the Na^+^ concentration, with a detection sensitivity of 46.1 mV (R^2^= 0.9993) per decade of concentration.


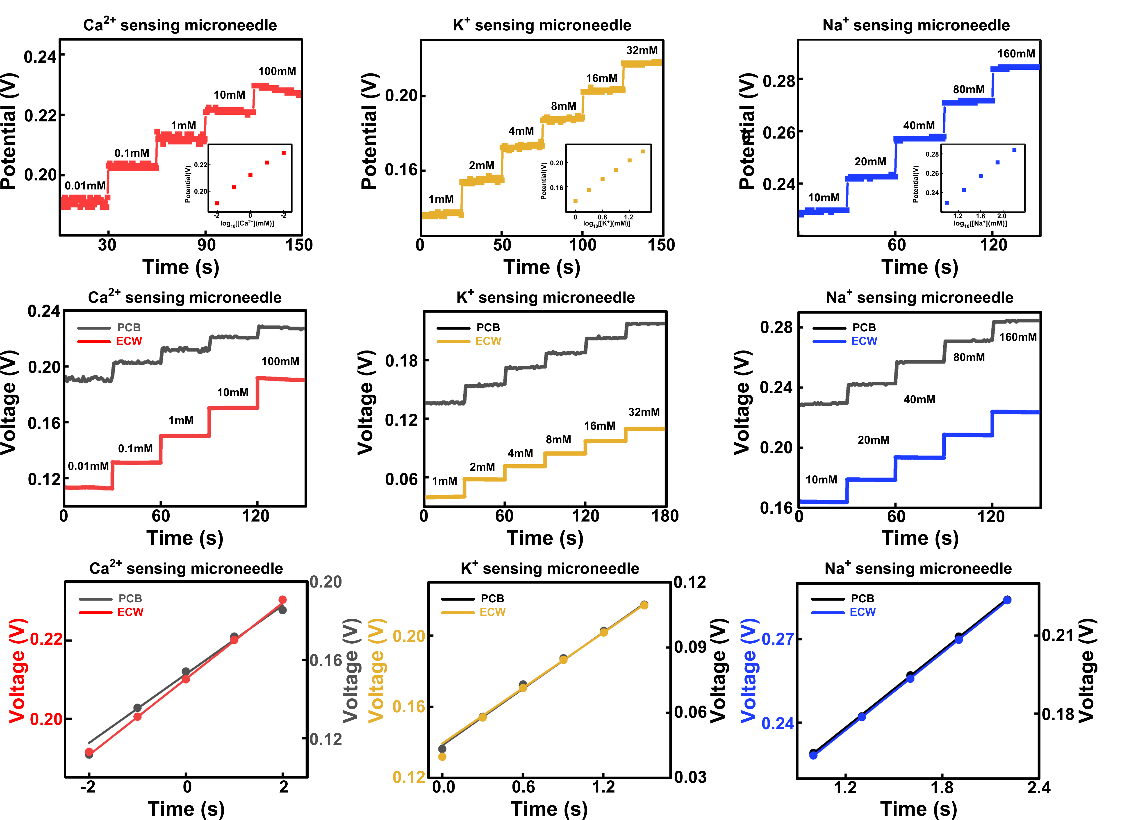


**Figure S17.** Graph showing the comparison of the voltage signals on ion-sensing electrodes using as-prepared PCB and commercial electrochemical workstation (ECW). Figures showed the different of the voltages and time using as-prepared PCB compared to ECW.

The potentiometric signal of the microneedle possessed good linearity with the Ca^2+^ concentration, with the detection sensitivity of 8.79 mV (R^2^= 0.9869) for as-prepared PCB and 19.49 mV (R^2^= 0.9990) for ECW per decade of concentration.

The potentiometric signal of the microneedle possessed good linearity with the K^+^ concentration, with the detection sensitivity of 52.9 mV (R^2^= 0.9977) for as-prepared PCB and 45.5 mV (R^2^= 0.9941) for ECW per decade of concentration.

The potentiometric signal of the microneedle possessed good linearity with the Na^+^ concentration, with the detection sensitivity of 46.1 mV (R^2^= 0.9993) for as-prepared PCB and 49.62 mV (R^2^= 0.9999) for ECW per decade of concentration.


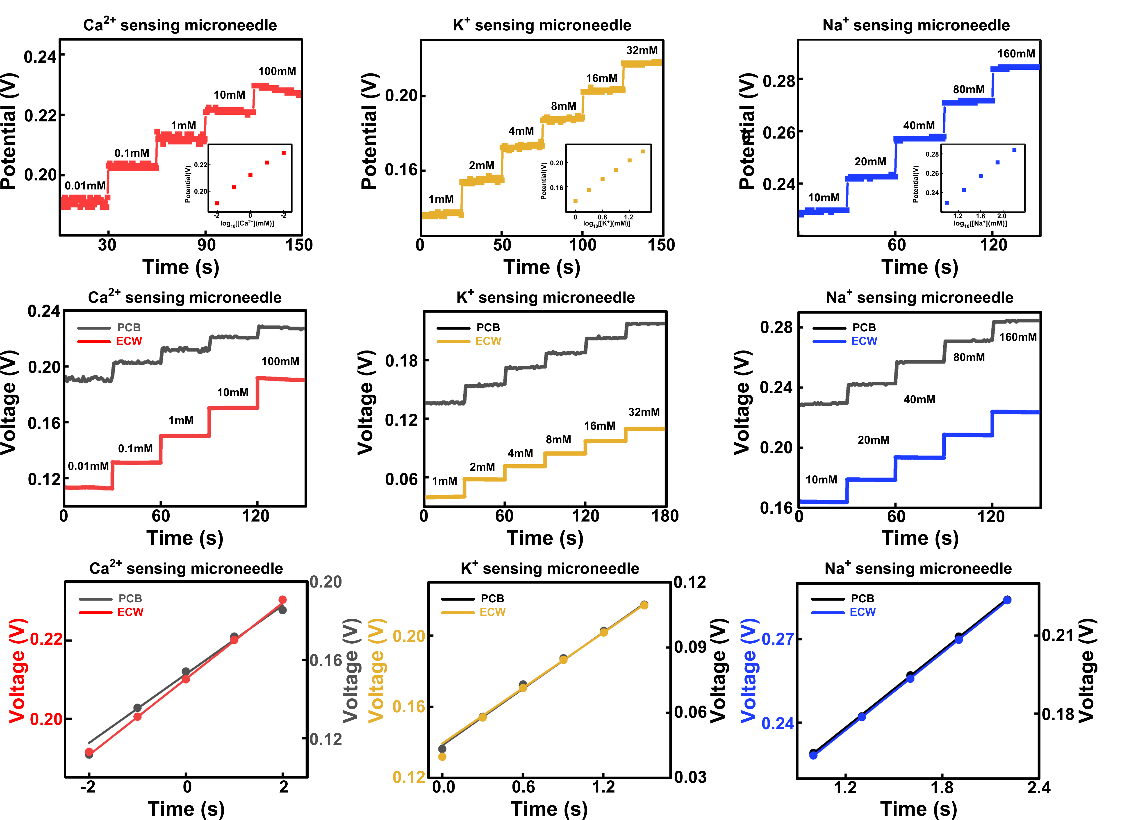


**Figure S18.** Graph showing the relations of the voltage signals on ion-sensing electrodes using as-prepared PCB and commercial electrochemical workstation (ECW). Figures showed the linear relations of the voltages and time using as-prepared PCB compared to ECW (with the black line).

The potentiometric signal of the microneedle possessed good linearity with the Ca^2+^ concentration, with the detection sensitivity of 8.79 mV (R^2^= 0.9869) for as-prepared PCB and 19.49 mV (R^2^= 0.9990) for ECW per decade of concentration.

The potentiometric signal of the microneedle possessed good linearity with the K^+^ concentration, with the detection sensitivity of 52.9 mV (R^2^= 0.9977) for as-prepared PCB and 45.5 mV (R^2^= 0.9941) for ECW per decade of concentration.

The potentiometric signal of the microneedle possessed good linearity with the Na^+^ concentration, with the detection sensitivity of 46.1 mV (R^2^= 0.9993) for as-prepared PCB and 49.62 mV (R^2^= 0.9999) for ECW per decade of concentration.


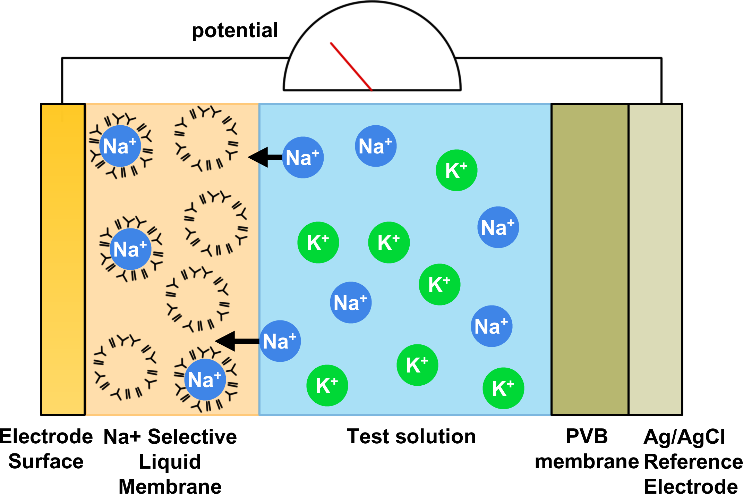


**Figure S19.** Graph showing the sensing principle of the ion-sensing electrode. Selective electrode was a potential sensor based on measuring the potential response related to the selective identification of a target ion analytes. Using a two-electrode system, the change in ion concentration was reflected by measuring the potential change (voltage difference) between the working electrode and reference electrode. A critical material in the potentiometric sensor was an electrode coated with a membrane that selectively allow passage of only one ionic species that will dominate the voltage signal.

Taking the Na^+^ sensor as an example, the PVC film coating embedded with Na^+^ probe -X will selectively allow only Na^+^ in the detection environment to pass through, then they will enter the electrode surface, be converted into electrons in PEDOT: PSS, and flowed into the inside of the electrode, resulting in potential change. As for non-target ions such as Ca^2+^, K^+^, Mg^2+^ and other interfering substances, they were blocked by the ion-selective membrane thus unable to produce potential changes. According to the Nernst's equation, the voltage depends on the logarithm of the ion activity (proportional to the ion concentration). With the increase of ion concentration in the solution, the number of ions diffusing into the membrane and the electrode increases, resulting in greater potential change.


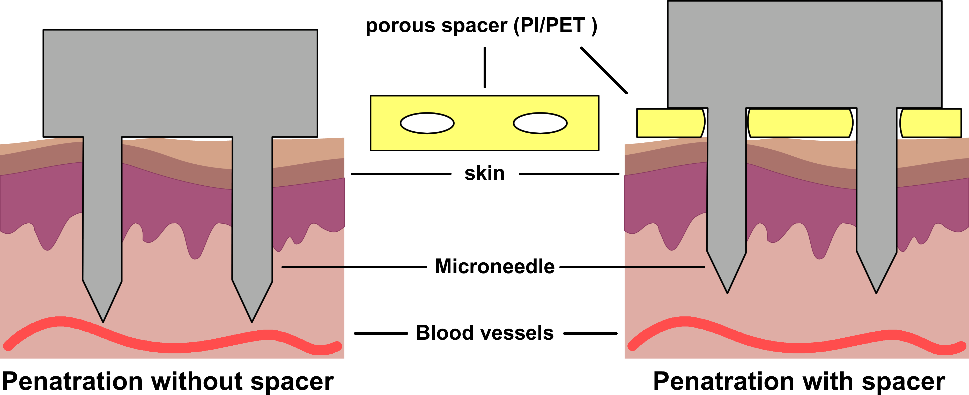


**Figure S20.** Graph showing that the depth of penetration of the microneedles was modulated by a porous insulator with a certain thickness. In this work, we designed the microneedles with a length of about 680 μm, which can penetrate the skin smoothly. In addition, when performed in animals, the tip of the microneedle was not fully inserted under the skin due to the curved tissue structure of the rat back, and part of the microneedle (~ 200 μm) was usually retained outside the skin. In the experiment, it was observed that the microneedle tips could contact the subcutaneous tissue fluid for sensing, but did not sting the skin and cause pain. Furthermore, the length of microneedles could be flexibly tuned by integrating a porous spacer (with certain thickness), such as a polyimide (PI) or polyethylene glycol terephthalate (PET) substrate (as showed in the schematic). and the length of the microneedle penetrating the skin can be effectively regulated.
